# Structural Engineering of Hierarchical Magnetic/Carbon Nanocomposites via In Situ Growth for High-Efficient Electromagnetic Wave Absorption

**DOI:** 10.1007/s40820-024-01396-3

**Published:** 2024-04-15

**Authors:** Xianyuan Liu, Jinman Zhou, Ying Xue, Xianyong Lu

**Affiliations:** Key Laboratory of Bio-Inspired Smart Interfacial Science and Technology of Ministry of Education, School of Chemistry, Beihang University, Beijing, 100191 People’s Republic of China

**Keywords:** Electromagnetic wave absorption, Hierarchical structure, In situ growth, Self-reduction

## Abstract

Materials exhibiting high-performance electromagnetic wave absorption have garnered considerable scientific and technological attention, yet encounter significant challenges. Developing new materials and innovative structural design concepts is crucial for expanding the application field of electromagnetic wave absorption. Particularly, hierarchical structure engineering has emerged as a promising approach to enhance the physical and chemical properties of materials, providing immense potential for creating versatile electromagnetic wave absorption materials. Herein, an exceptional multi-dimensional hierarchical structure was meticulously devised, unleashing the full microwave attenuation capabilities through in situ growth, self-reduction, and multi-heterogeneous interface integration. The hierarchical structure features a three-dimensional carbon framework, where magnetic nanoparticles grow in situ on the carbon skeleton, creating a necklace-like structure. Furthermore, magnetic nanosheets assemble within this framework. Enhanced impedance matching was achieved by precisely adjusting component proportions, and intelligent integration of diverse interfaces bolstered dielectric polarization. The obtain Fe_3_O_4_-Fe nanoparticles/carbon nanofibers/Al-Fe_3_O_4_-Fe nanosheets composites demonstrated outstanding performance with a minimum reflection loss (RL_min_) value of − 59.3 dB and an effective absorption bandwidth (RL ≤  − 10 dB) extending up to 5.6 GHz at 2.2 mm. These notable accomplishments offer fresh insights into the precision design of high-efficient electromagnetic wave absorption materials.

**Supplementary Information:**

The online version contains supplementary material available at 10.1007/s40820-024-01396-3.

## Highlights

- Hierarchical Fe_3_O_4_-Fe@CNFs/Al-Fe_3_O_4_-Fe nanocomposites were constructed by in situ growth, vacuum-assisted filtration, and self-reduction methods.
- The carbon framework, with in situ grown magnetic nanoparticles, supports two-dimensional magnetic nanosheets, achieving excellent electromagnetic performance and good impedance matching.
- Excellent reflection loss value (− 59.3 dB), broadband wave absorption (5.6 GHz at 2.2 mm thickness), and low radar cross-section value were achieved.

**Supplementary Information:**

The online version contains supplementary material available at 10.1007/s40820-024-01396-3.

## Graphical abstract

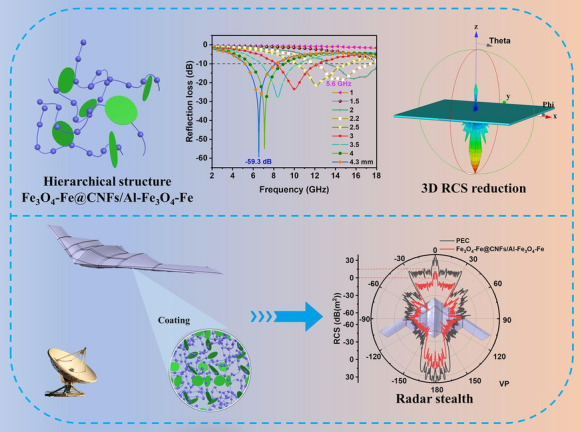

## Introduction

Materials engineered for electromagnetic wave (EMW) absorption have garnered significant interest due to their diverse applications in wireless communication, information processing, and radar stealth technology [1–4]. Moreover, these materials play a crucial role in addressing electromagnetic radiation pollution, which poses threats to human health and disrupts the operation of precision electronic equipment [5–7]. However, traditional EMW absorption materials encounter challenges in meeting the evolving needs of the high-tech era. Therefore, there is an urgent need to develop high-performance EMW absorption materials characterized by strong impedance matching, lightweight, small thickness, and a wide effective absorption band [8–10]. Over the past few decades, researchers have explored various material categories such as carbon-based materials, magnetic metals and their metal oxides or carbides, and semiconductor materials as potential candidates for EMW absorption [11, 12]. Based on the above results, combining magnetic/carbon elements with specialized structures can achieve efficient EMW absorption performance [13–15]. Many efforts have been made in expanding the application disciplines of EMW absorbers through the discovery of new materials and innovative structural design concepts. Strategic design involving reasonable hierarchical heterogeneous structures and zero-dimensional/one-dimensional/two-dimensional (0D/1D/2D) collaboration to induce multiple interfacial polarization and provide ample voids for scattering microwaves emerges as an effective approach in preparing high-performance EMW absorption materials [16, 17].

Artificial engineered structures of materials have captured significant interest in the past two decades due to their properties not found in natural materials for achieving efficient EMW absorption [18]. The creation of a hierarchical structure through the artificial arrangement of structural units represents an innovative approach aimed at regulating microstructures through organized spatial stacking [19, 20]. Designing special structures with appropriate electromagnetic parameters emerges as a viable strategy to enhance microwave absorption performance [21–23]. Aerogels, owing to their controllable composition and structure, serve as an excellent framework for constructing hierarchical structures. In addition, according to transmission line theory, the reflection loss (RL) values of the samples can be calculated. RL values are important indicators for directly assessing EMW absorption capability. Wu et al. [9] employed a top-down ice template method to prepare a series of MXenes aerogels with variable structures, encompassing disordered, porous, and lamellar structures. This approach simultaneously achieves the integration of electromagnetic stealth, thermal insulation, and load-carrying capacity. Wu et al. [24] introduced ordered heterostructure engineering by assembling MXenes/Aramid nanofibers/FeCo@SiO_2_ aerogel. This transformation converts disordered magnetic compositions into an ordered array, enhancing EMW absorption properties. Che et al. [25] fabricated a 3D hierarchical MoS_2_/FeS_2_ composite with minimum reflection loss value of -60.2 dB and an extended effective absorption bandwidth (EAB) to 6.48 GHz. Drawing inspiration from the exceptional microwave absorption properties of these unique structures, exploring new hierarchical structures with adjustable electromagnetic parameters holds promise for further enhancing EMW absorption performance.

Deftly engineering specialized structures with tailored electromagnetic parameters represents a viable strategy for enhancing their absorption performance of electromagnetic waves. Particularly, a novel special hierarchical structure combining 0D, 1D, and 2D materials is more attractive. The branched nanofibers derived from partial ionizing aramid microfibers offer versatility in constructing various 3D structures. The resulting branched aramid nanofibers (ANFs) easily undergo gelation, leading to the production of porous 3D aerogels [26]. Upon pyrolysis, ANFs aerogels yield a 3D continuous carbon network skeleton that supports other substances, contributing to dielectric and conductive losses. In the fabrication of composite materials, in situ growth proves to be a valuable method, addressing issues such as uneven mixing and weak binding forces between filler and matrix. This novel approach provides a conducive growth environment for inorganic materials to nucleate and grow in situ on the surface of organic materials. Challenges arise, when balancing the dielectric and magnetic loss capacities of in situ grown iron oxide nanoparticles on the 3D continuous aramid nanonetwork skeleton after carbonization, there are problems in impedance optimization and absorption performance of EMW. To overcome these challenges, reintroducing element-doped iron oxide into the composites and forming multi-dimensional, multi-heterogeneous interface absorption materials through autocatalytic pyrolysis emerges as a solution. Therefore, hierarchical materials characterized by low density, multi-dimensional components, tunable high dielectric loss, high specific surface area, and good impedance matching, undoubtedly represent a highly promising class of EMW absorption materials.

In this study, we employed an innovative approach encompassing in situ growth, vacuum-assisted filtration (VAF), and self-reducing calcination to fabricate hierarchical EMW absorption materials, demonstrating significant promise for practical applications. The process commenced with polyvinylpyrrolidone (PVP) serving as active nucleation sites on ANFs, facilitating the hydrothermal reaction to synthesize necklace-like *α-*Fe_2_O_3_@ANFs. Subsequently, the film was obtained by VAF of Al-*α*-Fe_2_O_3_ nanosheets and *α-*Fe_2_O_3_@ANFs mixture, which was then calcined to yield 0D/1D/2D Fe_3_O_4_-Fe nanoparticles/carbon nanofibers/Al-Fe_3_O_4_-Fe nanosheets (Fe_3_O_4_-Fe@CNFs/Al-Fe_3_O_4_-Fe) nanocomposites. The EMW absorption properties of these composites were fine-tuned by adjusting the amount of Al-*α*-Fe_2_O_3_ nanosheets. Carbon nanofibers (CNFs) provided a 3D network skeleton supporting magnetic Fe_3_O_4_-Fe nanoparticles and Al-Fe_3_O_4_-Fe nanosheets, resulting in a 3D hierarchical heterostructure. This hierarchical configuration featured a continuous carbon network for improved conduction loss, a rich internal cavity enhancing impedance matching, and facilitating robust interfacial polarization between magnetic Fe_3_O_4_-Fe nanoparticles and the surface of CNFs. The combined effects of these components resulted in adjusted electromagnetic parameters, improved impedance matching, and efficient EMW absorption performance. Detailed investigations on morphology, composition, structure, electromagnetic properties, EMW absorption characteristics, and the absorption mechanism underscore the potential significance of hierarchical multi-dimensional structures in advancing high-performance EMW absorption devices.

## Experimental Section

### Materials

Kevlar 49 fibers (diameter 1420 D, length 6338 m kg^−1^, density 1.44 g cm^−3^, degradation temperature 482 °C, elongation at break 2.46%) were purchased from DuPont (Wilmington, DE, USA). Dimethyl sulfoxide (DMSO) was supplied by Shanghai Aladdin Biochemical Technology Co., Ltd (Shanghai, China). Aluminum chloride (AlCl_3_), ferric nitrate nonahydrate (Fe(NO_3_)_3_·9H_2_O), sodium oxalate and triethylamine, ammonium acetate (CH_3_COONH_4_), polyvinylpyrrolidone (PVP, M_n_ = 5800), potassium hydroxide (KOH), and absolute ethanol were all provided by Shanghai Macklin Biochemical Co., Ltd (Shanghai, China). All these chemicals were used without further purification.

### Synthesis of ***α***-Fe_2_O_3_@ANFs

**Fig. 1:**
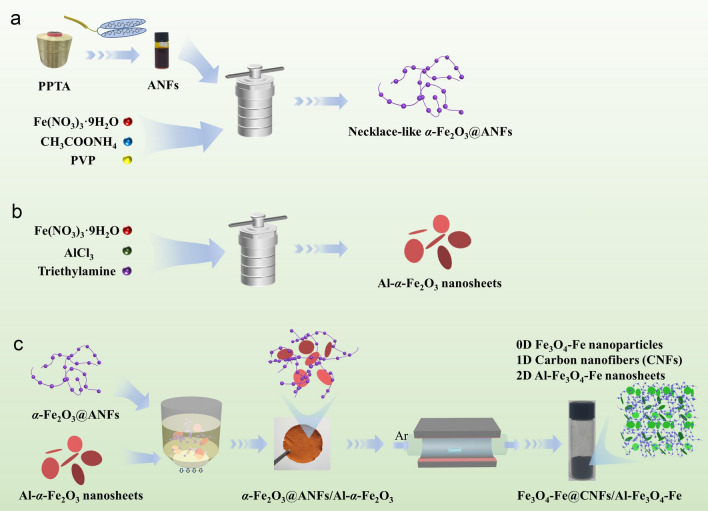
Fabricating process of **a** *α*-Fe_2_O_3_@ANFs, **b** Al-*α*-Fe_2_O_3_ nanosheets, and **c** Fe_3_O_4_-Fe@CNFs/Al-Fe_3_O_4_-Fe nanocomposites

Firstly, the aramid nanofibers (ANFs) solution was prepared according to a previous report [26]. 1.0 g of para-aramid fiber (Kevlar) was dispersed in 150 mL of DMSO, followed by the addition of 1.5 g of KOH and stirring for 7 days to obtain a dark red para-aramid nanofiber solution (ANFs solution). Subsequently, 45 mL of DMSO and 160 mL of deionized water were slowly added to 15 mL of ANFs solution under stirring, and the mixture was stirred for 24 h to obtain ANFs dispersion. The dispersion was centrifugal washed several times with deionized water and then re-dispersed into water to form 40 mL of ANFs dispersion. In this dispersion, 0.242 g of Fe(NO_3_)_3_·9H_2_O, 0.139 g of CH_3_COONH_4_, and 1.0 g of PVP were dissolved and magnetically stirred at 50 °C for 10 min. The mixture underwent ultrasonic dispersion at 100 W for 20 min. The resulting solution was transferred to a 100-mL Teflon-lined autoclave and reacted at 160 °C for 24 h. After the reaction, the product was centrifugal washed with deionized water and re-dispersed into water to form *α-*Fe_2_O_3_@ANFs dispersion. The corresponding preparation process is illustrated in Fig. 1a.

### Synthesis of Al-***α***-Fe_2_O_3_ Nanosheets

Al-*α*-Fe_2_O_3_ nanosheets were synthesized following a reported method [27]. Initially, 0.100 g of AlCl_3_ and 1.212 g of Fe(NO_3_)_3_·9H_2_O were dissolved in 30 mL of deionized water with magnetic stirring to form a transparent solution, while stirring, 4.5 mL of triethylamine was added, and the mixture was stirred for 40 min. The resulting solution was then transferred to a 100-mL Teflon-lined autoclave and reacted at 160 °C for 24 h. After the reaction, the red precipitate was collected through centrifugation and washed with deionized water several times. Subsequently, the synthesized ultra-thin Al-*α*-Fe_2_O_3_ nanosheets were dispersed in water, achieving a concentration of 24 mg mL^−1^ for further use. The corresponding preparation process is illustrated in Fig. 1b.

### Synthesis of Fe_3_O_4_-Fe@CNFs/Al-Fe_3_O_4_-Fe Nanocomposites

The *α*-Fe_2_O_3_@ANFs dispersion and 60 mL of Al-*α-*Fe_2_O_3_ solution were mixed and ultrasonically dispersed at 100 W for 20 min. The resulting dispersion was vacuum-filtered to fabricate a wet free-standing composited membrane, which was subsequently dried using supercritical CO_2_. The composite aerogel membrane was finally annealed at 700 °C for 4 h in an Ar atmosphere with a heating rate of 5 °C min^−1^, resulting in Fe_3_O_4_-Fe nanoparticles/carbon nanofibers/Al-Fe_3_O_4_-Fe nanosheets (Fe_3_O_4_-Fe@CNFs/Al-Fe_3_O_4_-Fe) nanocomposites. Additionally, products prepared with 20, 40, and 50 mL of Al-*α*-Fe_2_O_3_ solution were denoted as Fe_3_O_4_-Fe@CNFs/Al-Fe_3_O_4_-Fe_(1:1)_, Fe_3_O_4_-Fe@CNFs/Al-Fe_3_O_4_-Fe_(1:2)_, Fe_3_O_4_-Fe@CNFs/Al-Fe_3_O_4_-Fe_(1:2.5)_, respectively. The corresponding preparation process is illustrated in Fig. 1c.

### Characterization

The morphologies and size of the samples were characterized by scanning electron microscope (SEM) using JSM-7500F (JEOL, Japan) with an operating voltage of 5 kV. Transmission electron microscope (TEM) images was carried out on FEI Tecnai F20 at an acceleration voltage of 200 kV. For TEM samples, the procedure entails dispersing the appropriate amount of sample into ethanol, subjecting it to ultrasonic dispersion at 800 W for 1 min using a pressure cell disruptor, dropping the mixture onto a copper mesh, drying, and then conducting TEM analysis. X-ray diffraction (XRD) patterns were obtained using Ultima IV (Rigaku, Japan) in the 2*θ* range of 10–80° at scanning rate of 2° min^−1^. The hysteresis loops were performed by vibrating sample magnetometer (VSM) using LakeShore7404 (Lake Shore, USA). Thermogravimetric analysis (TGA) of the samples was carried by STA8000 (PerkinElmer, USA) from 25 to 800 °C at a heating rate of 10 °C/min under a nitrogen atmosphere. X-ray photoelectron spectra (XPS) of the samples was carried out on K-Alpha (Thermo Scientific, USA). The complex permittivity and permeability of the samples were determined using E5071C (Agilent, USA) vector network analyzer according the coaxial-line theory in a frequency range of 2–18 GHz. The absorbent materials were prepared by mixing the sample powder into paraffin matrix with a weight of 30 wt%. The mixture was then pressed into a circular ring (outer diameter: 7.00 mm, inner diameter: 3.04 mm, thickness: ~ 2.00 mm).

## Results and Discussion

### Preparation and Characterization of Fe_3_O_4_-Fe@CNFs/Al-Fe_3_O_4_-Fe

**Fig. 2:**
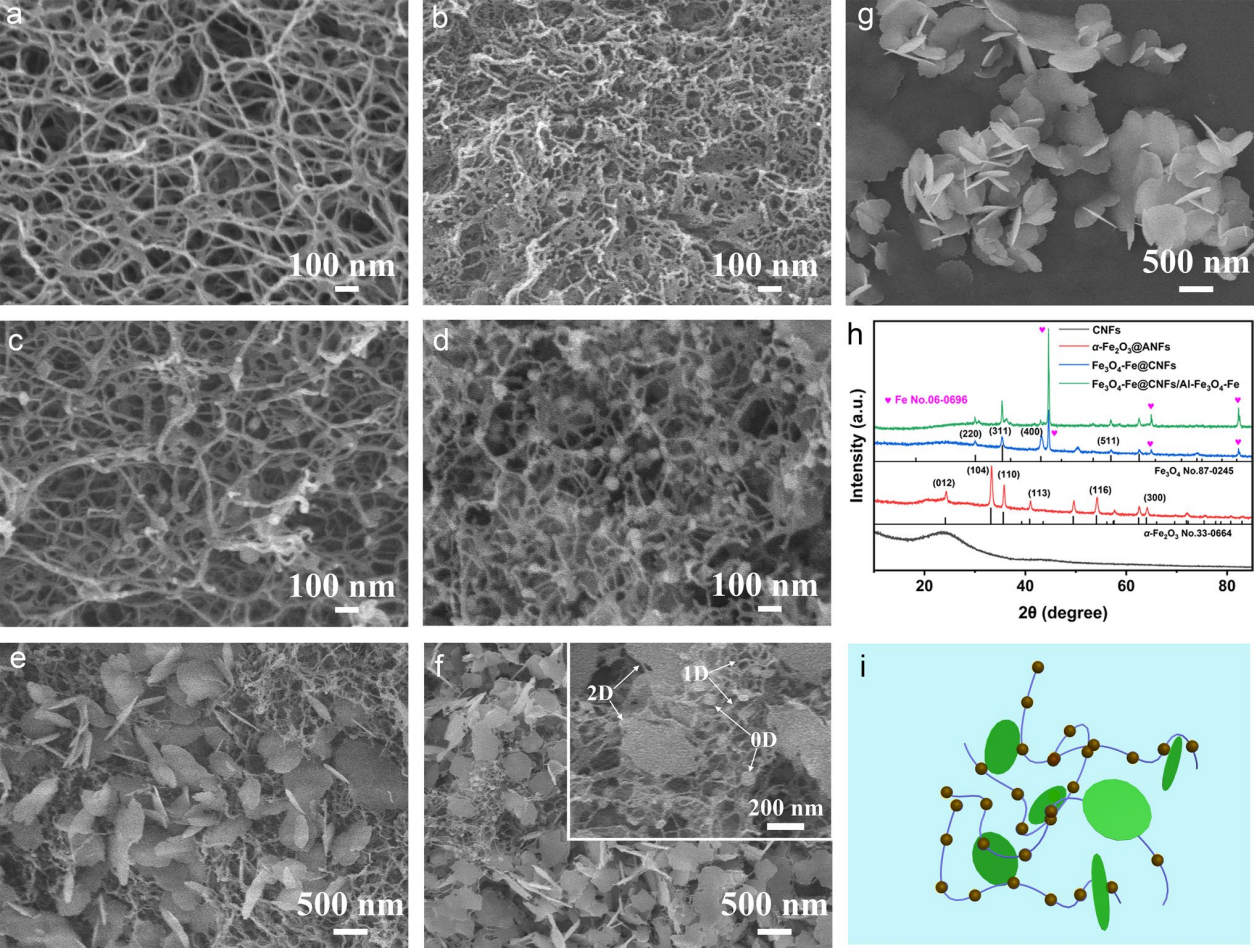
SEM images of **a** ANFs aerogel, **b** CNFs, **c** *α*-Fe_2_O_3_@ANFs aerogel, **d** Fe_3_O_4_-Fe@CNFs, **e** *α-*Fe_2_O_3_@ANFs/Al-*α*-Fe_2_O_3_ aerogel, **f** Fe_3_O_4_-Fe@CNFs/Al-Fe_3_O_4_-Fe, and **g** Al-*α*-Fe_2_O_3_ nanosheets. **h** XRD patterns of CNFs, *α*-Fe_2_O_3_@ANFs, Fe_3_O_4_-Fe@CNFs, and Fe_3_O_4_-Fe@CNFs/Al-Fe_3_O_4_-Fe. **i** Structure diagram of Fe_3_O_4_-Fe@CNFs/Al-Fe_3_O_4_-Fe

The preparation procedure of the Fe_3_O_4_-Fe@CNFs/Al-Fe_3_O_4_-Fe nanocomposites is delineated in Fig. 1. Initially, the aramid nanofiber solution was generated through the deprotonation of micron-scale aramid fibers (PPTA), leading to the breakage of hydrogen bonds and the formation of a polymer solution. Water was subsequently introduced to reconstruct hydrogen bonds, leading to the dispersion of ANFs. The necklace-like *α*-Fe_2_O_3_@ANFs was synthesized through an in situ growth process of *α*-Fe_2_O_3_ on ANFs via a facile hydrothermal reaction, as illustrated in Fig. 2c. In this reaction, PVP was utilized to establish catalytically active sites on the surface of ANFs, thereby facilitating the nucleation and in situ growth of *α*-Fe_2_O_3_ nanoparticles. The composite film was generated through VAF of the Al-*α*-Fe_2_O_3_ nanosheets and *α*-Fe_2_O_3_@ANFs dispersions, and the composite aerogel (*α*-Fe_2_O_3_@ANFs/Al-*α*-Fe_2_O_3_) was successfully fabricated via supercritical drying with CO_2_ (Fig. 2e). The optical image of the surface of the *α*-Fe_2_O_3_@ANFs/Al-*α*-Fe_2_O_3_ aerogel is illustrated in Fig. S1a. The aerogels, denoted as *α*-Fe_2_O_3_@ANFs/Al-*α*-Fe_2_O_3(1:1, 1:2, 1:2.5)_, exhibit similar morphologies (Fig. S1b–d). Furthermore, ANFs aerogel alone exhibits a 3D network structure by the supercritical dry, as depicted in Fig. 2a. Subsequently, the *α*-Fe_2_O_3_@ANFs/Al-*α*-Fe_2_O_3_ aerogel underwent high-temperature calcination, yielding in 0D/1D/2D Fe_3_O_4_-Fe@CNFs/Al-Fe_3_O_4_-Fe nanocomposites.

Following high-temperature calcination, ANFs transform into CNFs, maintaining the integral 3D network structure (Fig. 2b). The *α*-Fe_2_O_3_@ANFs was transformed into Fe_3_O_4_-Fe@CNFs after exposure to high temperature, maintaining 3D network appearance (Fig. 2d). The ANFs in *α*-Fe_2_O_3_@ANFs act as a skeleton, effectively separating *α*-Fe_2_O_3_ and preventing extensive ceramic sintering. The Al-*α*-Fe_2_O_3_ nanosheets exhibits an ultra-thin 2D structure (Fig. 2g). Agglomerative sintering was observed when Al-*α*-Fe_2_O_3_ nanosheets alone were calcined into Al-Fe_3_O_4_ nanosheets in a reducing atmosphere (Fig. S2a). The Fe_3_O_4_-Fe@CNFs/Al-Fe_3_O_4_-Fe nanocomposites present a 3D network structure, with 0D Fe_3_O_4_-Fe nanoparticles, 1D carbon nanofibers, and 2D Al-Fe_3_O_4_-Fe nanosheets clearly visible (Fig. 2f), contributing to excellent electromagnetic wave (EMW) absorption performance in multiple dimensions. Al-Fe_3_O_4_-Fe was affixed to the Fe_3_O_4_-Fe@CNFs skeleton, forming a 3D aerogel structure. With decreasing Al-Fe_3_O_4_-Fe content in Fe_3_O_4_-Fe@CNFs/Al-Fe_3_O_4_-Fe_(1:1, 1:2, 1:2.5)_, morphological deformation and pores emerge attributed to ceramic sintering (Fig. S2b-d). The phases, structures, and components of samples were further analyzed by XRD (Fig. 2h). The primary diffraction peaks at 24.3°, 33.2°, 35.8°, 41.0°, 54.2°, and 64.1° for *α*-Fe_2_O_3_@ANFs correspond to the crystal planes (012), (104), (110), (113), (116), and (300) of *α*-Fe_2_O_3_ (JCPDS card No. 33–0664), respectively. Similarly, the primary diffraction peaks at 30.1°, 35.4°, 43.1°, and 57.0° for Fe_3_O_4_-Fe@CNFs and Fe_3_O_4_-Fe@CNFs/Al-Fe_3_O_4_-Fe correspond to the crystal planes (220), (311), (400), and (511) of Fe_3_O_4_ (JCPDS card No. 87–0245), respectively. Notably, there are also 44.6°, 64.9°, and 82.3° correspond to the crystal planes (110), (200), and (211) of Fe (JCPDS card No. 06–0696) in Fe_3_O_4_-Fe@CNFs and Fe_3_O_4_-Fe@CNFs/Al-Fe_3_O_4_-Fe. The major diffraction peaks align with crystal faces *α*-Fe_2_O_3_ and Fe_3_O_4_. *α*-Fe_2_O_3_@ANFs/Al-*α*-Fe_2_O_3_ was calcined at 700 °C for 4 h to form Fe_3_O_4_-Fe@CNFs/Al-Fe_3_O_4_-Fe, with Ar atmosphere during calcination. No reducing gas was introduced during calcining process of Fe_3_O_4_-Fe@CNFs/Al-Fe_3_O_4_-Fe nanocomposites, and the nanocomposites underwent self-reduction through their own components. ANFs in the *α*-Fe_2_O_3_@ANFs/Al-*α*-Fe_2_O_3_ were transformed into carbon material CNFs in an inert gas, while CNFs react with *α*-Fe_2_O_3_ in the nanocomposites, reducing it to Fe_3_O_4_, and some Fe_3_O_4_ was further reduced to Fe. The XRD pattern in Fig. 2h can provide additional insights into this self-reduction process. For comparison, Al-*α*-Fe_2_O_3_ nanosheets were reduced to Al-Fe_3_O_4_ nanosheets. XRD patterns of Al-*α*-Fe_2_O_3_ and Al-Fe_3_O_4_ nanosheets are depicted in Fig. S3. Therefore, the Fe_3_O_4_-Fe@CNFs/Al-Fe_3_O_4_-Fe nanocomposites is a hierarchical structure composed of Fe_3_O_4_-Fe nanoparticles, CNFs, and Al-Fe_3_O_4_-Fe nanosheets. The corresponding structure diagram is shown in Fig. 2i.

**Fig. 3:**
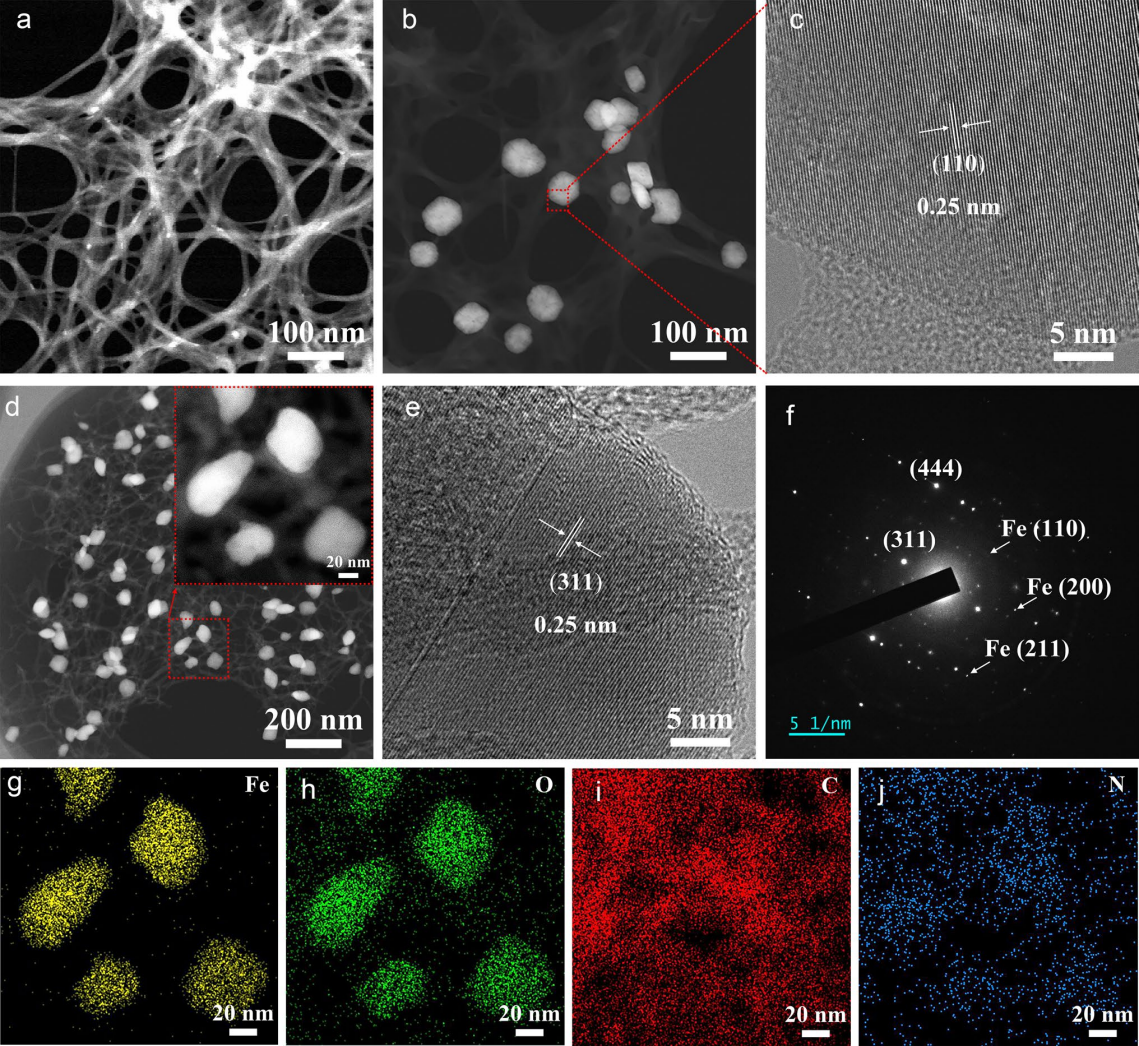
STEM images of **a** ANFs, **b** *α*-Fe_2_O_3_@ANFs, and **d** Fe_3_O_4_-Fe@CNFs/Al-Fe_3_O_4_-Fe. **c** High-resolution TEM of *α*-Fe_2_O_3_ in *α*-Fe_2_O_3_@ANFs. **e** High-resolution TEM image of Fe_3_O_4_-Fe in Fe_3_O_4_-Fe@CNFs/Al-Fe_3_O_4_-Fe and its corresponding **f** SAED image. **g-j** Elemental mapping images of Fe, O, C, N in Fe_3_O_4_-Fe@CNFs/Al-Fe_3_O_4_-Fe

PPTA transformed to ANFs with a 3D network structure, as illustrated in Fig. 3a. The surface of ANFs was subsequently modified by constructing catalytic active sites using PVP, which selectively adsorbed on the ANFs surface. Then, *α-*Fe_2_O_3_ nanoparticles were grown in situ at the catalytic site through a hydrothermal reaction (Fig. 3b). The locally magnified high-resolution TEM (Fig. 3c) clearly shows a crystal plane spacing of 0.25 nm, consistent with the (110) crystal plane of *α*-Fe_2_O_3_. Figure S4a illustrates the TEM image of *α*-Fe_2_O_3_@ANFs in other region, where the sample exhibits a necklace-like structure, and particles tightly bind to fibers. After calcination, the volume of Fe_3_O_4_-Fe@CNFs has contracted, resulting in finer fibers (Fig. S4b). The TEM image of Fe_3_O_4_-Fe@CNFs/Al-Fe_3_O_4_-Fe in the corresponding region (Fig. S5) displays 0D, 1D, and 2D components, aligning with the SEM image (Fig. 2f). The hierarchical heterogeneous structure of 0D Fe_3_O_4_-Fe nanoparticles, 1D carbon nanofibers, and 2D Al-Fe_3_O_4_-Fe nanosheets (Fe_3_O_4_-Fe@CNFs/Al-Fe_3_O_4_-Fe) was formed, as shown in Fig. 3d. It clearly shows the necklace-like structure of Fe_3_O_4_-Fe nanoparticles attached to CNFs. The corresponding element mapping in Fig. 3g-j and XRD patterns in Fig. 2h confirm the presence of Fe_3_O_4_-Fe nanoparticles in the composites, with distributed C and N elements in the samples. The N element originates from aramid fiber, and after calcination, ANFs transform into CNFs, resulting in N-doped carbon that significantly enhances the EMW absorption performance of the materials [28]. In the high-resolution TEM image of Fe_3_O_4_-Fe nanoparticles in Fe_3_O_4_-Fe@CNFs/Al-Fe_3_O_4_-Fe (Fig. 3e), the (311) crystal faces with a crystal face spacing of 0.25 nm are clearly visible. The selected area electron diffraction (SAED) image in Fig. 3f indicates that Fe_3_O_4_ is a single crystal with (311) and (444) crystal face spots. Additionally, the SAED image shows Fe (110), (200), and (211) crystal face spots, confirming the reduction of some Fe_3_O_4_ to Fe.

**Fig. 4:**
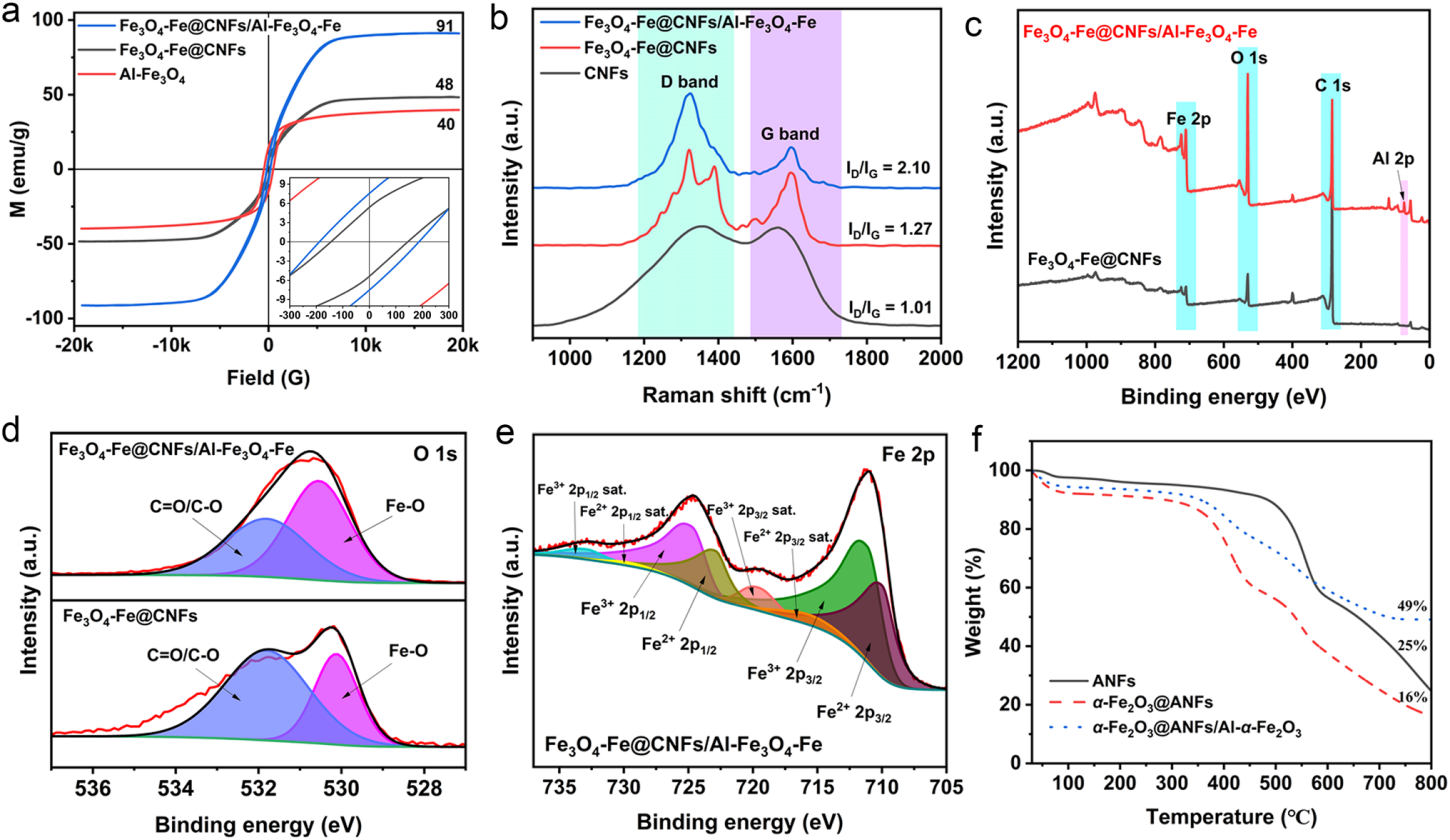
**a** Magnetic hysteresis loops of Fe_3_O_4_-Fe@CNFs nanoparticles, Al-Fe_3_O_4_ nanosheets, and Fe_3_O_4_-Fe@CNFs/Al-Fe_3_O_4_-Fe. **b** Raman spectra of CNFs, Fe_3_O_4_-Fe@CNFs particles, and Fe_3_O_4_-Fe@CNFs/Al-Fe_3_O_4_-Fe. XPS spectra of **c** survey scan and **d** O 1*s* of Fe_3_O_4_-Fe@CNFs, and Fe_3_O_4_-Fe@CNFs/Al-Fe_3_O_4_-Fe. **e** XPS spectra of Fe 2*p* of Fe_3_O_4_-Fe@CNFs/Al-Fe_3_O_4_-Fe. **f** TGA curves of ANFs, *α*-Fe_2_O_3_@ANFs, and *α*-Fe_2_O_3_@ANFs/Al-*α*-Fe_2_O_3_

The magnetic properties of as-synthesized of Fe_3_O_4_@CNFs, Al-Fe_3_O_4_ nanosheets, and Fe_3_O_4_-Fe@CNFs/Al-Fe_3_O_4_-Fe nanocomposites were measured by vibrating sample magnetometer (VSM), as illustrated in Fig. 4a. Necklace-like Fe_3_O_4_@CNFs, Al-Fe_3_O_4_ nanosheets, and Fe_3_O_4_-Fe@CNFs/Al-Fe_3_O_4_-Fe nanocomposites exhibit superparamagnetic behavior with saturation magnetization of 48, 40, and 91 emu g^−1^, respectively. The high saturation magnetization of Fe_3_O_4_-Fe@CNFs/Al-Fe_3_O_4_-Fe is attributed to Fe nanocrystals existing in Al-Fe_3_O_4_-Fe (Fig. 2h). Additionally, the presence of carbon defects in the samples was studied through Raman spectra (Fig. 4b). In general, the D band arises from defect/disordered carbons, and the G band is attributed to in-plane stretching vibration of *sp*^2^ carbons [28, 29]. Therefore, the *I*_D_*/I*_G_ ratio of as-prepared nanocomposites can be used to characterize the defect degree of carbon components by Raman spectra. In Fig. 4b, CNFs, Fe_3_O_4_-Fe@CNFs, and Fe_3_O_4_-Fe@CNFs/Al-Fe_3_O_4_-Fe have *I*_D_/*I*_G_ values of 1.01, 1.27, and 2.10, respectively. Fe_3_O_4_-Fe@CNFs/Al-Fe_3_O_4_-Fe shows the highest *I*_D_/*I*_G_ due to Fe_3_O_4_-Fe and Al-Fe_3_O_4_-Fe promoting carbon defects, favoring EMW absorption [28]. In addition, the chemical structures and surface electronic states of Fe_3_O_4_-Fe@CNFs, and Fe_3_O_4_-Fe@CNFs/Al-Fe_3_O_4_-Fe were analyzed by XPS. As shown in Fig. 4c, the XPS spectra of Fe_3_O_4_-Fe@CNFs and Fe_3_O_4_-Fe@CNFs/Al-Fe_3_O_4_-Fe show the presence of Fe, O and C, while Fe_3_O_4_-Fe@CNFs/Al-Fe_3_O_4_-Fe has obvious Al element signal. In the O 1*s* spectrum (Fig. 4d), there are chemical bonds between the iron and oxygen atoms, corresponding to Fe–O and C=O/C–O bonds at 530.3 and 531.8 eV, respectively. In Fig. 4e, the high-resolution Fe 2*p* spectrum of Fe_3_O_4_-Fe@CNFs/Al-Fe_3_O_4_-Fe can be deconvolved into eight peaks. Peaks at 711.0 and 724.5 eV correspond to Fe 2*p*_3/2_ and Fe 2*p*_1/2_ of Fe^3+^, while peaks at 709.7 and 722.5 eV represent Fe 2*p*_3/2_ and Fe 2*p*_1/2_ of Fe^2+^, and other peaks are satellite peaks resulted from shake-up excitation of high-spin oxidized Fe species [30]. These results confirm the presence of Fe_3_O_4_, not γ-Fe_2_O_3_. XPS spectra of Fe 2*p* of Fe_3_O_4_-Fe@CNFs can be divided into peaks same as Fe_3_O_4_-Fe@CNFs (Fig. S6). To characterize the transition from *α*-Fe_2_O_3_@ANFs/Al-*α*-Fe_2_O_3_ to Fe_3_O_4_-Fe@CNFs/Al-Fe_3_O_4_-Fe, the samples were analyzed by thermogravimetric analysis (Fig. 4f). In an inert gas atmosphere, pure ANFs decompose most rapidly at 556 °C, resulting in the decomposition of aramid nanofibers into carbon materials CNFs. The corresponding differential thermogravimetric (DTG) curves of samples is shown in Fig. S7. The decomposition curves of *α*-Fe_2_O_3_@ANFs and α-Fe_2_O_3_@ANFs/Al-*α*-Fe_2_O_3_ exhibit peaks around 410 and 550 °C, where the former is attributed to the decomposition of PVP in the system, while the latter is due to ANFs decomposition. The decomposition of aramid fibers into carbon materials CNFs, facilitates the reduction of *α*-Fe_2_O_3_ to Fe_3_O_4_ and Fe. The heat resistance of nanocomposites increases with Al-*α*-Fe_2_O_3_ introduction, which indicates *α*-Fe_2_O_3_@ANFs/Al-*α*-Fe_2_O_3_ nanocomposites have a higher residual weight ratio at 800 °C than pure ANFs. The introduction of inorganic materials can further improve the heat resistance of polymers [31, 32]. It can better elucidate this process of the nanocomposites combining the XRD in Fig. 2h and the VSM in Fig. 4a. In this process, one component in the samples acts as a reducing agent, enabling the reduction in other components, hence termed as the self-reduction process of nanocomposites.

### EMW Absorption Performance

**Fig. 5:**
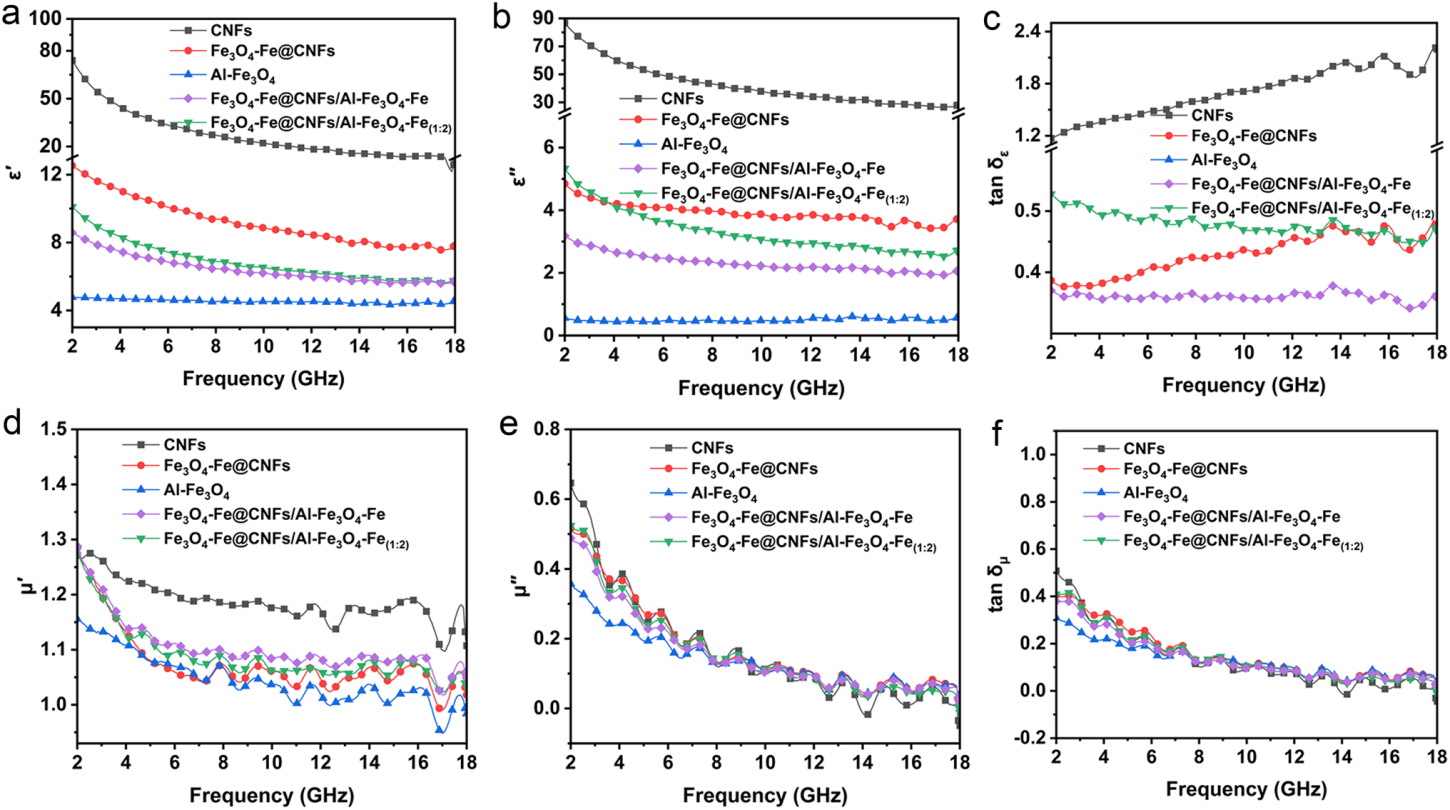
Frequency dependence of **a** the real part (*ε*ʹ) and **b** imaginary part (*εʺ*) of the complex permittivity, **c** dielectric loss tangent, **d** the real part (*μ*ʹ) and **e** imaginary part (*μʺ*) of the complex permeability, and **f** magnetic loss tangent of CNFs, Fe_3_O_4_-Fe@CNFs, Al-Fe_3_O_4_, Fe_3_O_4_-Fe@CNFs/Al-Fe_3_O_4_-Fe, and Fe_3_O_4_-Fe@CNFs/Al-Fe_3_O_4_-Fe_(1:2)_

The relative complex permittivity (*ε*_*r*_ = *εʹ* − *jεʺ*), relative complex permeability (*μ*_*r*_ = *μʹ* − *jμʺ*), dielectric loss and magnetic loss of CNFs, Fe_3_O_4_-Fe@CNFs nanoparticles, Al-Fe_3_O_4_ nanosheets, Fe_3_O_4_-Fe@CNFs/Al-Fe_3_O_4_-Fe, and Fe_3_O_4_-Fe@CNFs/Al-Fe_3_O_4_-Fe_(1:2)_ are depicted in Fig. 5. The real parts of dielectric constant (*εʹ*) and permeability (*μʹ*) reflect the electrical and magnetic energy storage capacity of the materials, while the imaginary parts (*εʺ* and *μʺ*) indicate energy loss [33]. A higher tan *δ*_*ε*_ value implies better electrical energy absorption capability [34, 35]. CNFs exhibit significantly higher dielectric constant and tan *δ*_*ε*_ than other materials, due to the excellent conductivity of CNFs, allowing electrons to freely propagate within them. However, CNFs lack magnetic loss, limiting their EMW absorption performance. Magnetic loss is theoretically divided into three mechanisms (exchange resonance, natural resonance, and eddy current loss) [9]. Introducing magnetic Fe_3_O_4_-Fe nanoparticles into CNFs can significantly reduce their dielectric real and imaginary parts, but they remain at a relatively high level, which is detrimental to EMW absorption. By adjusting the proportions of magnetic and conductive components, the composite material’s electromagnetic parameters are fine-tuned to achieve impedance matching. Further introducing low-dielectric Al-Fe_3_O_4_-Fe nanosheets into the nanocomposites can additionally reduce the complex permittivity of the composites, and adjusting the Al-Fe_3_O_4_-Fe nanosheets content maintains appropriate dielectric properties. Figure 5a illustrates that as the Al-Fe_3_O_4_-Fe nanosheets content increases, the real part of the dielectric constant of the nanocomposites decreases, thus adjusting the EMW absorption performance. The wide scattering area of 2D Al-Fe_3_O_4_-Fe nanosheets enhances EMW attenuation. The dielectric real parts of CNFs, Fe_3_O_4_-Fe@CNFs nanoparticles, Al-Fe_3_O_4_ nanosheets, Fe_3_O_4_-Fe@CNFs/Al-Fe_3_O_4_-Fe, and Fe_3_O_4_-Fe@CNFs/Al-Fe_3_O_4_-Fe_(1:2)_ exhibit a gradual decrease at high frequencies, which is associated with the dispersion effects present in the samples [21]. In Fig. 5d, *μʹ* values decrease with frequency, which may be due to relaxation of the magnetic moment process rather than hysteresis. From Fig. 4d, e, it is evident that the magnetic permeability parameters of the samples fluctuate, which is related to the occurrence of natural resonance within the samples. In Fe_3_O_4_-Fe@CNFs/Al-Fe_3_O_4_-Fe nanocomposites, 0D Fe_3_O_4_-Fe nanoparticles, 1D CNFs, and 2D Al-Fe_3_O_4_-Fe nanosheets optimize impedance matching for excellent EMW absorption.

**Fig. 6:**
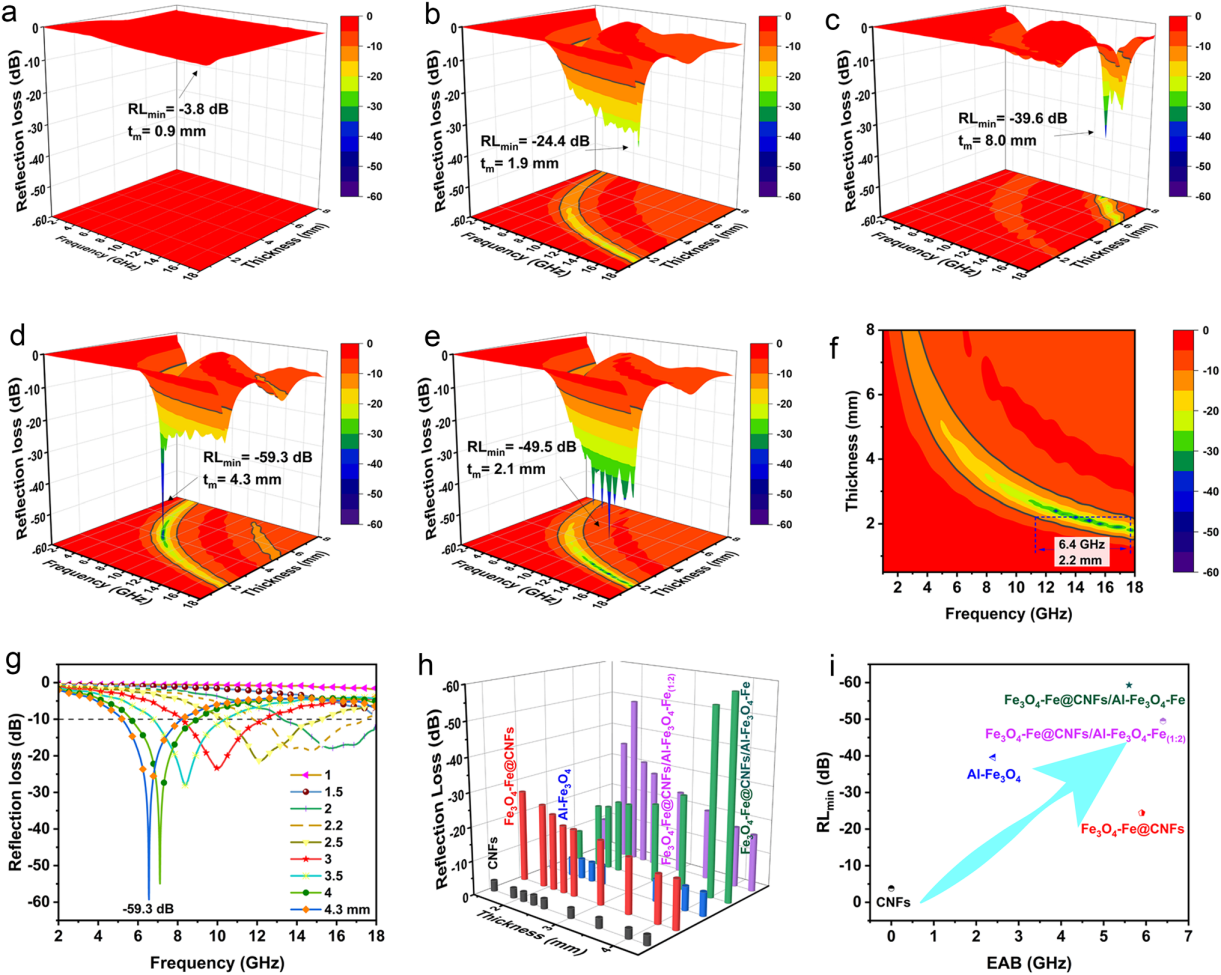
3D reflection loss curves maps of **a** CNFs, **b** Fe_3_O_4_-Fe@CNFs, **c** Al-Fe_3_O_4_, **d** Fe_3_O_4_-Fe@CNFs/Al-Fe_3_O_4_-Fe, and **e** Fe_3_O_4_-Fe@CNFs/Al-Fe_3_O_4_-Fe_(1:2)_. **f** 2D contour map of Fe_3_O_4_-Fe@CNFs/Al-Fe_3_O_4_-Fe_(1:2)_. **g** 2D reflection loss curve of Fe_3_O_4_-Fe@CNFs/Al-Fe_3_O_4_-Fe. **h** 3D plots of minimum reflection loss evaluation at different thicknesses of samples. **i** Comprehensive comparison on minimum reflection loss and effective absorption bandwidths of samples

Based on the transmission line theory [36, 37], the reflection loss can be calculated by the following formulas:

1 $$Z_{{{\text{in}}}} = Z_{0} \sqrt {\frac{{\mu_{r} }}{{\varepsilon_{r} }}} {\text{tan}}h\left[ {j\left( {\frac{2\pi fd}{c}} \right)\sqrt {\mu_{r} \varepsilon_{r} } } \right]$$

2 $${\text{RL}} = 20{\text{log}}\left| {\left( {Z_{{{\text{in}}}} - Z_{0} } \right)/\left( {Z_{{{\text{in}}}} + Z_{0} } \right)} \right|$$

where *Z*_in_ represents the normalized input impedance, *Z*_*0*_ is the impedance of free space, *ε*_*r*_ and *μ*_*r*_ are the relative complex permittivity and permeability, respectively, *f* is the frequency of the microwave, *d* is the thickness of the absorber, and *c* is the velocity of light. In evaluating EMW absorbers, key criteria include minimum reflection loss (RL_min_) and effective absorption bandwidth (EAB) [38–40]. In practical applications, the EAB is the frequency range where the RL value is less than − 10 dB, which signifies the absorption of 90% of electromagnetic energy [41]. The EMW absorption properties of the samples are shown in Fig. 6. Their minimum reflection losses, effective absorption bandwidths, and corresponding thicknesses are listed in Table S1. Notably, Fe_3_O_4_-Fe@CNFs/Al-Fe_3_O_4_-Fe has RL_min_ of − 59.3 dB (6.6 GHz) at 4.3 mm and an EAB of 5.6 GHz (11.8–17.4 GHz) at 2.2 mm (Fig. 6d). Fe_3_O_4_-Fe@CNFs/Al-Fe_3_O_4_-Fe_(1:2)_ shows RL_min_ of − 49.5 dB (14.9 GHz) at 2.1 mm and an EAB of 6.4 GHz (11.3–17.7 GHz) at 2.2 mm (Fig. 6e). Additionally, the 3D reflection loss curves maps of Fe_3_O_4_-Fe@CNFs/Al-Fe_3_O_4_-Fe_(1:1)_ and Fe_3_O_4_-Fe@CNFs/Al-Fe_3_O_4_-Fe_(1:2.5)_ are presented in Fig. S8. Fe_3_O_4_-Fe@CNFs/Al-Fe_3_O_4_-Fe_(1:2)_ offers thickness and EAB advantages compared to Fe_3_O_4_-Fe@CNFs/Al-Fe_3_O_4_-Fe, as depicted in the 2D contour map (Fig. 6f), while Fe_3_O_4_-Fe@CNFs/Al-Fe_3_O_4_-Fe has superior RL_min_, as shown in the 2D reflection loss curve (Fig. 6g). With the increase in Al-Fe_3_O_4_-Fe nanosheets content in Fe_3_O_4_-Fe@CNFs/Al-Fe_3_O_4_-Fe nanocomposites, the minimum reflection loss decreases, corresponding to an increase in thickness, and the effective absorption bandwidth initially increases and then decreases (Table S1). Therefore, the content of Al-Fe_3_O_4_-Fe in the composite materials plays a role in regulating the performance of the electromagnetic parameters, thus regulating the EMW absorption performance of the materials. Fe_3_O_4_-Fe@CNFs/Al-Fe_3_O_4_-Fe emerges as a more competitive option, offering significant advantages in terms of both EAB and RL_min_ values when compared to other samples, as depicted in Fig. 6h-i. These results underscore the importance of carbon and magnetic components in electromagnetic wave (EMW) absorption. Table 1 offers a comparison of the EMW absorption capabilities of Fe_3_O_4_-Fe@CNFs/Al-Fe_3_O_4_-Fe with other materials mentioned in published studies. The RL_min_ of Fe_3_O_4_-Fe@CNFs/Al-Fe_3_O_4_-Fe at − 59.3 dB is stronger than other absorbing materials. Additionally, the EAB of Fe_3_O_4_-Fe@CNFs/Al-Fe_3_O_4_-Fe_(1:2)_ at 6.4 GHz is wider than other materials, and its thickness is also smaller. This is primarily attributed to the hierarchical structure of the composite material and the synergistic interaction among its components. The results demonstrate that Fe_3_O_4_-Fe@CNFs/Al-Fe_3_O_4_-Fe has certain advantages in terms of *RL*_min_, effective absorption bandwidth, and thickness.

**Table 1:** Comparison of this work with reported materials for EMW absorption

Absorbers	Shapes	RL_min_		RL ≤ − 10 dB		References
		RL_min_ (dB)	Thickness (mm)	EAB (GHz)	Thickness (mm)	
Fe_3_O_4_-graphene	Fibers	− 40.0	4.5	3.6	–	[42]
Ni@NC	Nanoflakes	− 37.1	2.3	6.2	2.3	[28]
Co@NCNTs/CF	Reticular fiber	− 57.8	4.0	4.5	2.0	[29]
CNF@C-Ni	nanofibers with flower	− 49.8	2.2	5.4	2.1	[43]
Co@NC	Dodecahedron	− 53.0	1.8	6.2	2.0	[44]
Fe_3_O_4_@CNTs	Necklace-like	− 51.3	4.4	3.9	–	[45]
Fe_3_O_4_-Fe@CNFs/Al-Fe_3_O_4_-Fe	Hierarchical structure	− 59.3	4.3	5.6	2.2	This work

*NC* N-doped carbon, *CF* carbon fiber, *CNF* carbon nanofiber, *CNTs* carbon nanotube

**Fig. 7:**
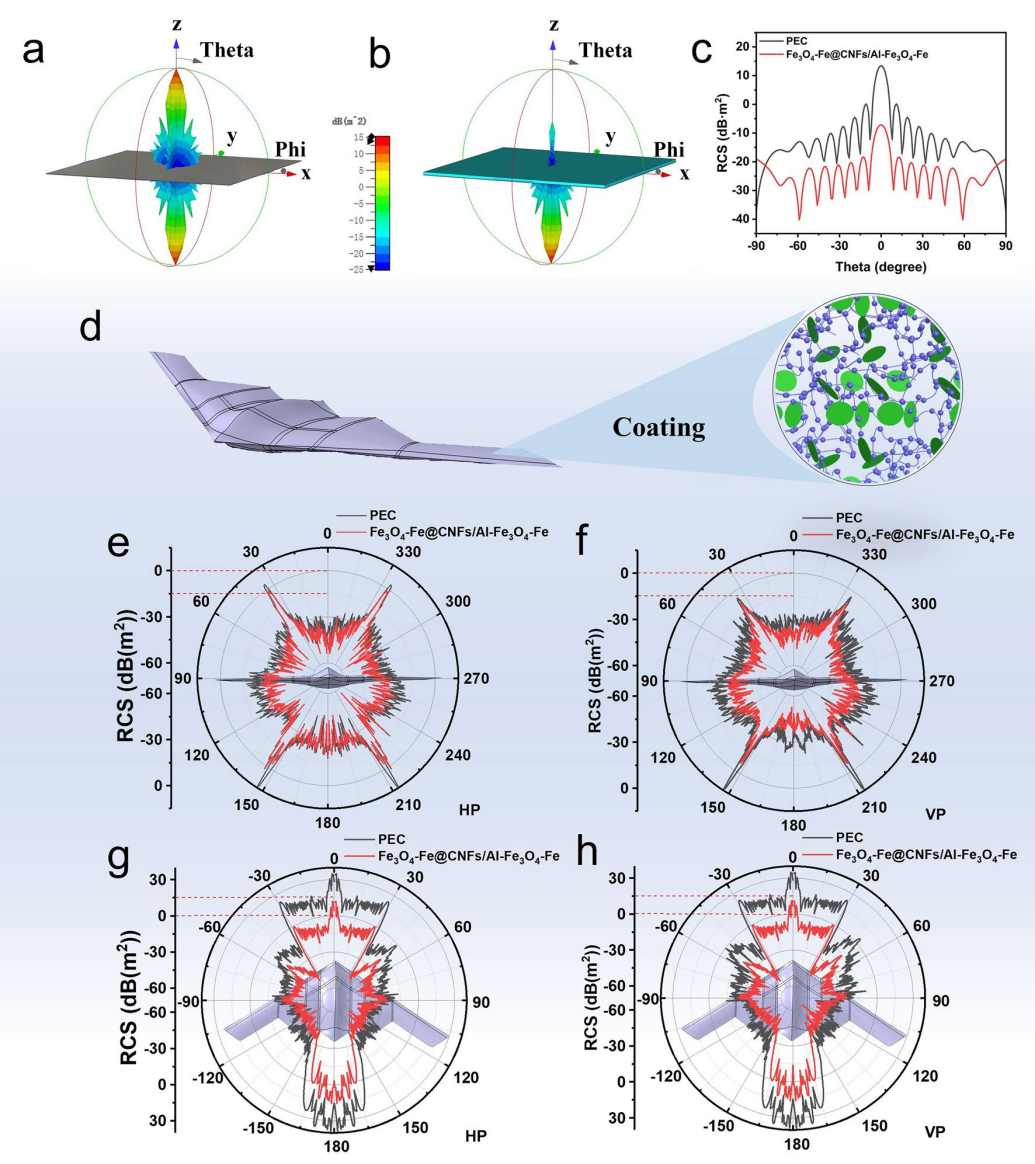
CST simulation results. 3D *RCS* plots for **a** PEC substrate and **b** PEC covered with Fe_3_O_4_-Fe@CNFs/Al-Fe_3_O_4_-Fe, size: 180 × 180 × 2.5 mm^3^. **c** Simulated *RCS* values of samples under certain detecting angles. **d** Airplane covered with Fe_3_O_4_-Fe@CNFs/Al-Fe_3_O_4_-Fe coating. Forward view *RCS* curve of the airplane at 10 GHz under **e** horizontal polarization and **f** vertical polarization. Top view *RCS* curve of the airplane at 10 GHz under **g** horizontal polarization and **h** vertical polarization

The radar cross-section (*RCS*) simulation is further carried out to evaluate the actual far-field EMW absorption performance. In general, the *RCS* value (*σ*) is determined by theta and phi in spherical coordinates, as shown below [46, 47]:

3 $$\sigma \left( {dBm^{2} } \right) = 10{\text{log}}\left[ {\frac{4\pi S}{{\lambda^{2} }}\left| {\frac{{E_{s} }}{{E_{i} }}} \right|^{2} } \right]$$

where *S, λ, E*_*s*_ and *E*_*i*_ are the area of the simulated plate, the length of the incident EMW, the scattered field intensity of transmitting waves, and the incident field intensity of receiving waves. In general, once the shape and geometric configuration of a device are determined, *RCS* data can assess the device’s EMW absorption and reflection capabilities. Due to the strong electromagnetic scattering affecting metal components of military targets, traditional equipment is easily detected by radar [48]. Coating military equipment surfaces with microwave-absorbing layers reduces *RCS* [49]. To evaluate the practical application potential of Fe_3_O_4_-Fe@CNFs/Al-Fe_3_O_4_-Fe, the *RCS* of a 180 × 180 × 0.5 mm^3^ perfect electrical conductor (PEC) plate substrate coated with a 2.5 mm thick Fe_3_O_4_-Fe@CNFs/Al-Fe_3_O_4_-Fe layer was simulated using CST STUDIO SUITE 2023 software. Here, positive x-axis is phi 0°, positive z-axis is theta 0°, the plate is placed on the *x-o-y* plane in the direction of positive z-axis, and the EMW enters the model along the z-axis. The 3D results (Fig. 7a, b) maintain incident angles of EMW phi = 0° and theta = 0° at 12.5 GHz, receiving results at all angles. The 2D result (Fig. 7c) maintains incident angles of EMW phi = 0° and theta = 0° at 12.5 GHz, receiving angle phi = 0° and adjusting theta from -90° to 90° for receiving. The 3D radar wave scattering signals from the original PEC plate and the Fe_3_O_4_-Fe@CNFs/Al-Fe_3_O_4_-Fe-coated plate at 12.5 GHz were examined. The scattered signal from the Fe_3_O_4_-Fe@CNFs/Al-Fe_3_O_4_-Fe-coated PEC plate is significantly lower than that of the PEC substrate (Fig. 7a-b), indicating that Fe_3_O_4_-Fe@CNFs/Al-Fe_3_O_4_-Fe has an effective absorption capacity. The 2D curve of simulated *RCS* values in the *x-o-z* plane (Fig. 7c) shows a drop in *RCS* values from 13.4 dB m^2^ (PEC) to 7.2 dB m^2^ (PEC coated with Fe_3_O_4_-Fe@CNFs/Al-Fe_3_O_4_-Fe) when both theta and phi are 0°. *RCS* simulation results demonstrate Fe_3_O_4_-Fe@CNFs/Al-Fe_3_O_4_-Fe’s ability to effectively suppress EMW scattering and reflection from the PEC substrate surface. For a better practical application characterization, Fe_3_O_4_-Fe@CNFs/Al-Fe_3_O_4_-Fe is coated on the surface of an airplane with a thickness of 3 mm (Fig. 7d), and EMW absorption effects are simulated. Here, positive x-axis is phi 0°, positive z-axis is theta 0°, and the airplane is placed on the *x-o-y* plane and the nose of the plane is facing the direction of positive x-axis. The direction of the incident EMW is a function of theta and phi. The forward view *RCS* curve of the airplane (receiving angles theta = 90°, phi = 0 ~ 360°) and the overview *RCS* curve (receiving angles theta = -180° ~ 180°, phi = 90°) at 10 GHz were tested for horizontal polarization (HP) and vertical polarization (VP). Compared to the PEC-based model, the airplane coated with Fe_3_O_4_-Fe@CNFs/Al-Fe_3_O_4_-Fe absorbers reflects much weaker signals (Fig. 7e-h). These simulation results highlight Fe_3_O_4_-Fe@CNFs/Al-Fe_3_O_4_-Fe composites promising applications in both civil and military stealth coatings.

### EMW Absorption Mechanism

**Fig. 8:**
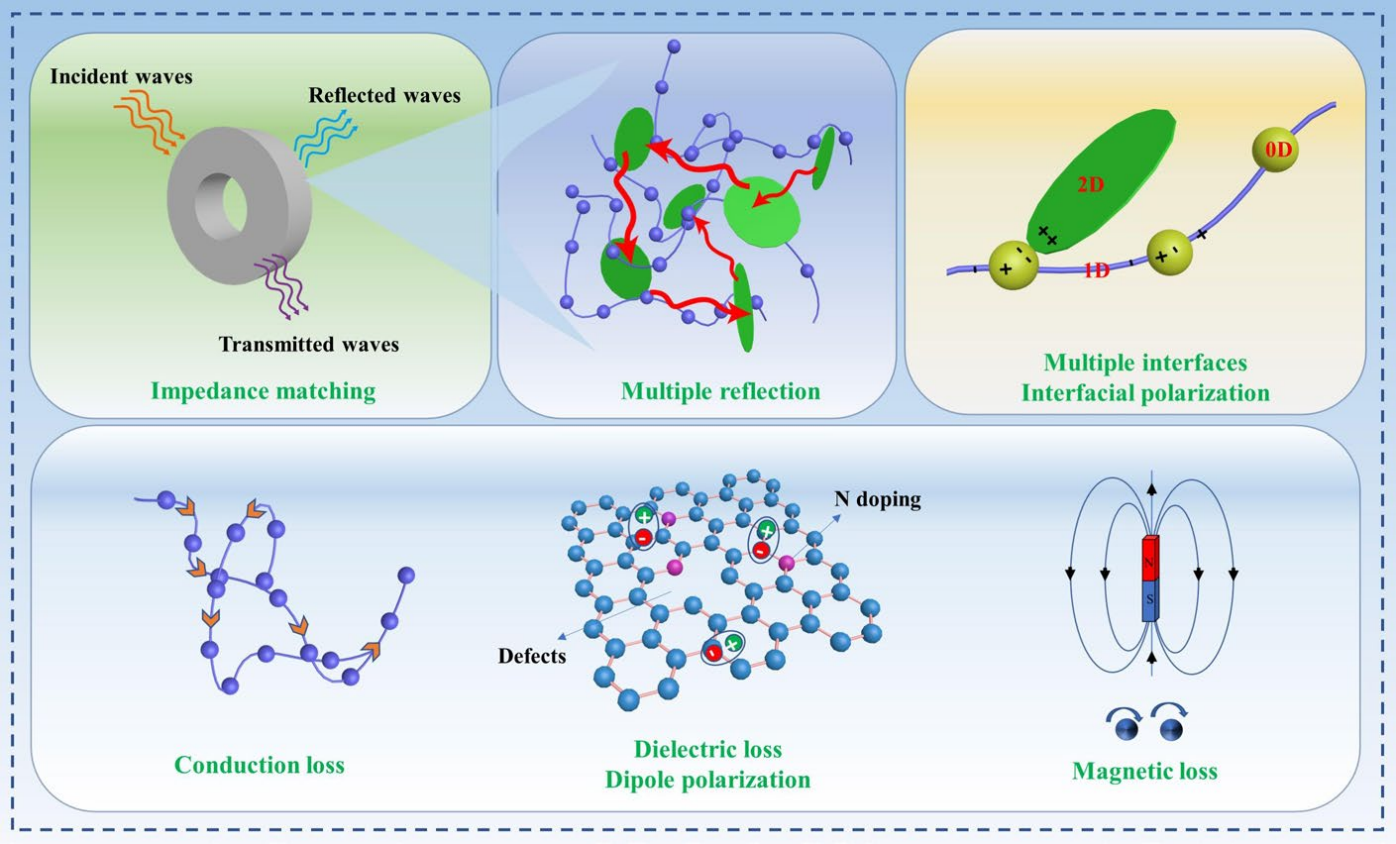
Schematic illustration of the microwave absorption mechanisms of the Fe_3_O_4_-Fe@CNFs/Al-Fe_3_O_4_-Fe

The possible EMW absorption mechanism of Fe_3_O_4_-Fe@CNFs/Al-Fe_3_O_4_-Fe is illustrated in Fig. 8. The exceptional EMW absorption performance primarily stems from the synergy of dielectric loss, conduction loss, and magnetic loss [50, 51]. In Fe_3_O_4_-Fe@CNFs/Al-Fe_3_O_4_-Fe nanocomposites, 1D CNFs play a crucial role as framework materials in forming the conductive network of the nanocomposites. On the other hand, CNFs with abundant defects, oxygen-containing functional groups, and dangling bonds provide active sites for dipole polarization loss [52]. For the 2D Al-Fe_3_O_4_-Fe nanosheets, electromagnetic waves are repeatedly scattered by the nanosheets upon entering the absorber, thereby increasing multiple scattering losses [53]. In addition, the content of Al-Fe_3_O_4_-Fe nanosheets can effectively control the conductivity of hierarchical nanocomposites and optimize the electromagnetic parameters of nanocomposite materials. As for the 0D Fe_3_O_4_-Fe nanoparticles, the heterogeneous interface between the nanoparticles and CNFs is conducive to interface polarization, thus improving dielectric loss. Additionally, the introduction of Fe_3_O_4_-Fe nanoparticles and Al-Fe_3_O_4_-Fe nanosheets can also provide magnetic losses, thereby improving impedance matching [54]. In addition, the Fe_3_O_4_-Fe nanoparticles and Al-Fe_3_O_4_-Fe nanosheets are mixed with Fe. Because Fe exhibits strong saturation magnetization, and its presence further enhances the saturation magnetization of Fe_3_O_4_-Fe@CNFs/Al-Fe_3_O_4_-Fe nanocomposites (Fig. 4a). Fe is present within Fe_3_O_4_, forming heterogeneous interfaces, resulting in interface polarization. Components at heterogeneous interfaces exhibit differences in conductivity, thus under the action of an electromagnetic field, free charges can be trapped at the interface, leading to the accumulation of positive and negative charges in the interface region [55]. In addition, the hierarchical structure, artificially designed, integrating 0D/1D/2D materials was developed. The prepared hierarchical Fe_3_O_4_-Fe@CNFs/Al-Fe_3_O_4_-Fe nanocomposites exhibit excellent EMW absorption performance, closely related to multi-dimensional gradient structure, multiple loss mechanisms, and synergistic effects of each component. Moreover, Fe_3_O_4_-Fe@CNFs/Al-Fe_3_O_4_-Feʹs 3D hierarchical heterogeneous structure provides multiple interfaces for interfacial polarization and ample voids for scattering microwaves. These factors enable the composites to achieve high-efficiency EMW absorption with a low filling load and thin thickness.

## Conclusions

New materials and innovative structural design concepts are of great significance to the application electromagnetic wave absorption. Constructing hierarchical heterostructures is an effective strategy for creating efficient electromagnetic wave absorption materials. Hierarchical Fe_3_O_4_-Fe@CNFs/Al-Fe_3_O_4_-Fe nanocomposites were prepared by employing in situ growth, VAF, and self-reduction calcination strategies. Fe_3_O_4_-Fe@CNFs/Al-Fe_3_O_4_-Fe achieves an excellent RL_min_ value of -59.3 dB at a thickness of 4.3 mm, with an EAB of 5.6 GHz at 2.2 mm. The 3D carbon skeleton establishes a continuous electrical and thermal conductivity network, with magnetic Fe_3_O_4_-Fe enhancing the material’s magnetic loss performance. Two-dimensional Al-Fe_3_O_4_-Fe nanosheets increase the multiple reflection of electromagnetic waves, and the presence of Fe in the components further enhances magnetic loss and interfacial polarization, ensuring efficient conversion of electromagnetic waves into heat dissipation. Additionally, the outstanding electromagnetic wave absorption performance of the nanocomposites is related to the multiple loss mechanisms, multi-dimensional gradient structures, and the synergistic effect of each component. The research findings provide important guidance and inspiration for the design of multilayer hierarchical structured materials in electromagnetic wave absorption materials and other related fields.

## Supplementary Information

Below is the link to the electronic supplementary material.

Supplementary file1 (DOCX 4557 kb)
