# Supplementary material for: Structural Engineering of Hierarchical Magnetic/Carbon Nanocomposites via In Situ Growth for High-Efficient Electromagnetic Wave Absorption
Source: Nanomicro Lett. 2024 Apr 15;16:174. doi: 10.1007/s40820-024-01396-3 (PMC11018581; doi:10.1007/s40820-024-01396-3)
Supplement: Supplementary file 1 — Supplementary file1 (DOCX 4557 kb) [file 40820_2024_1396_MOESM1_ESM.docx]

Supporting Information for

**Structural Engineering of** **Hierarchical Magnetic/Carbon Nanocomposites via In-Situ Growth for High-Efficient Electromagnetic Wave Absorption**

Xianyuan Liu^1^, Jinman Zhou^1^, Ying Xue, Xianyong Lu^1,^ *

Key Laboratory of Bio-Inspired Smart Interfacial Science and Technology of Ministry of Education, School of Chemistry, Beihang University, Beijing 100191, P. R. China

*Corresponding author. E-mail: [xylu@buaa.edu.cn](mailto:xylu@buaa.edu.cn) (Xianyong Lu)

**Supplementary Figures and Table**


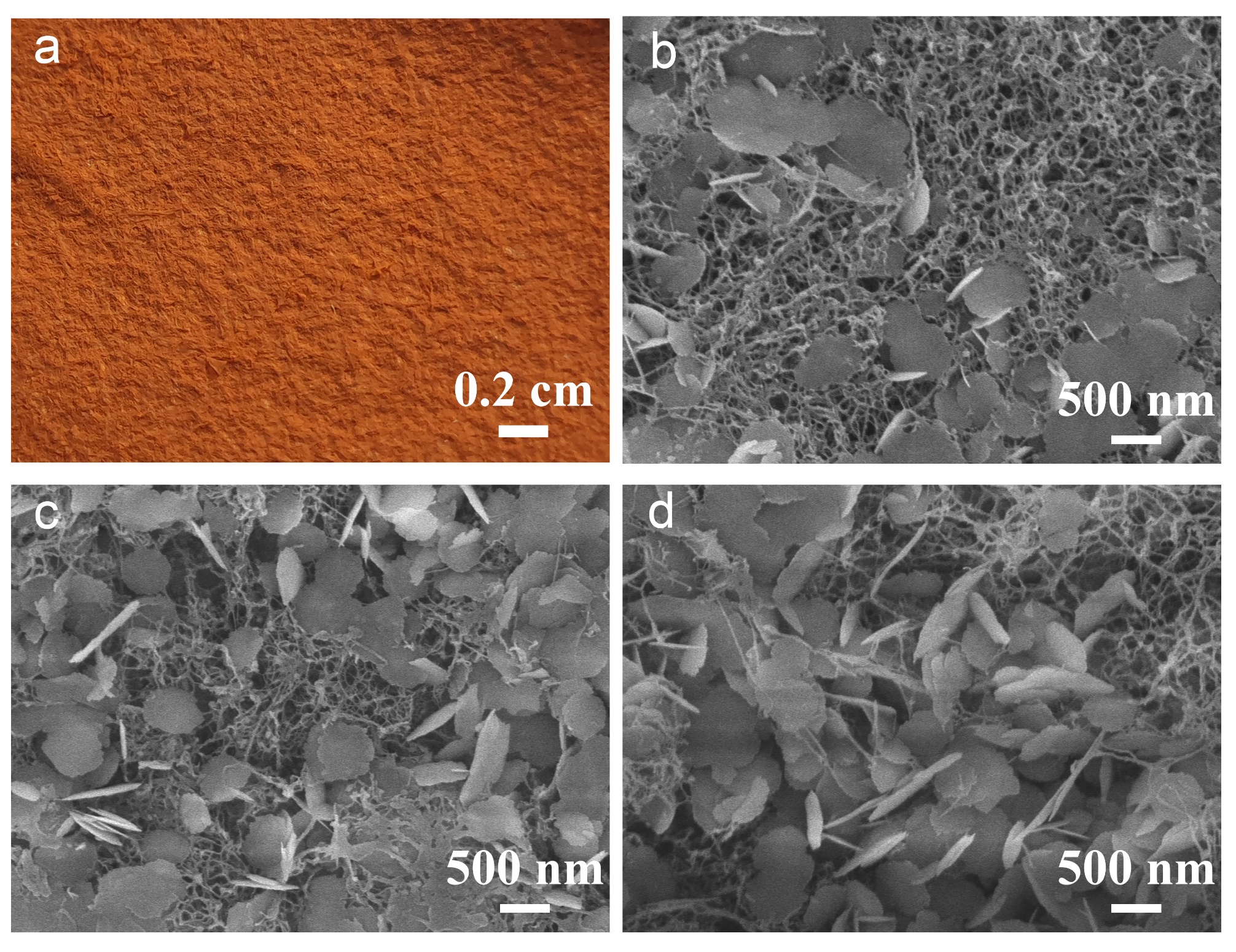


**Fig. S1** **a** Optical image of the surface of *α*-Fe_2_O_3_@ANFs/Al-*α*-Fe_2_O_3_ aerogel. SEM images of **b** *α*-Fe_2_O_3_@ANFs/Al-*α*-Fe_2_O_3(1:1)_ aerogel, **c** *α*-Fe_2_O_3_@ANFs/Al-*α*-Fe_2_O_3(1:2)_ aerogel, and **d** *α*-Fe_2_O_3_@ANFs/Al-*α*-Fe_2_O_3(1:2.5)_ aerogel


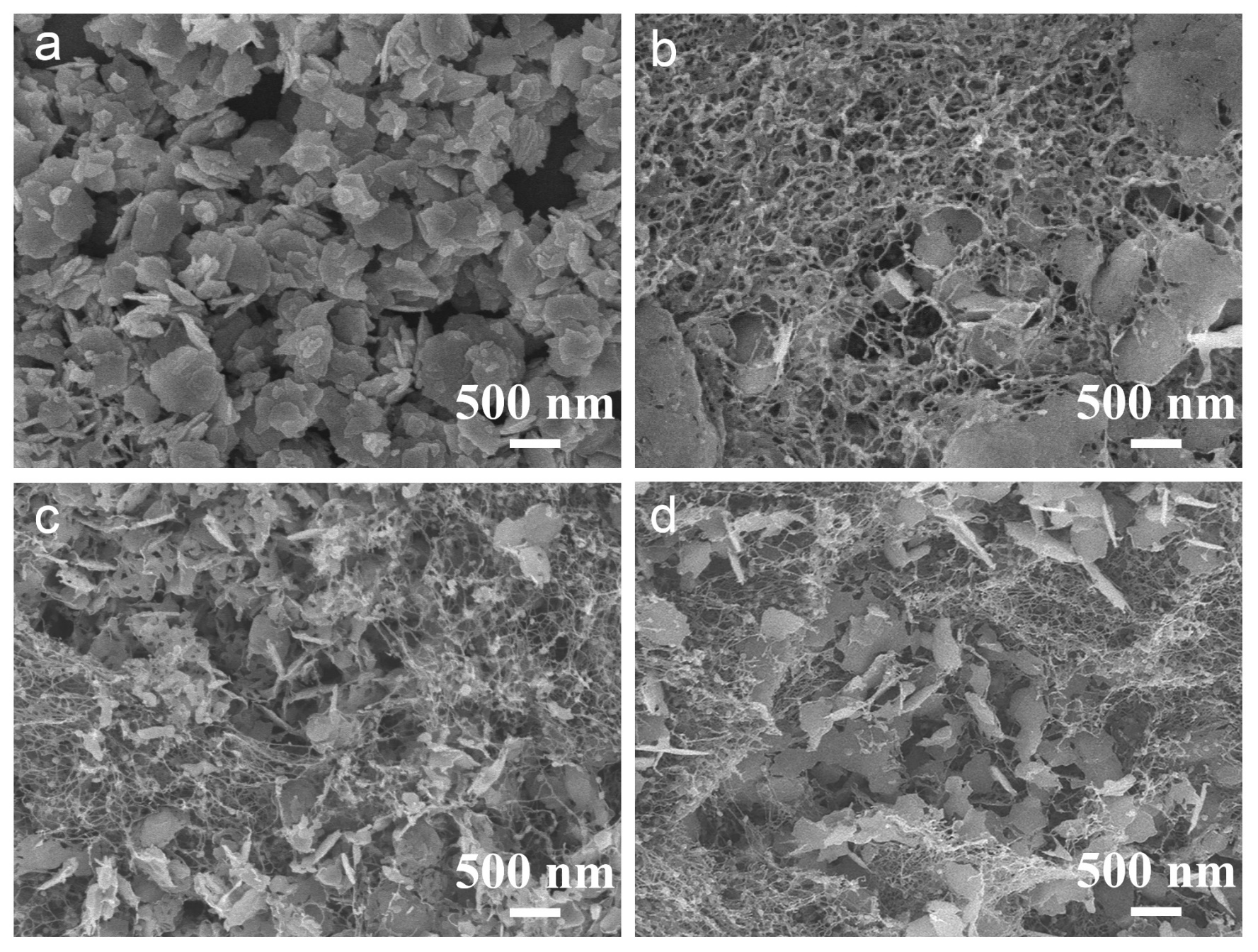


**Fig. S2** SEM images of **a** Al-Fe_3_O_4_ nanosheets, **b** Fe_3_O_4_-Fe@CNFs/Al-Fe_3_O_4_-Fe_(1:1)_, **c** Fe_3_O_4_-Fe@CNFs/Al-Fe_3_O_4_-Fe_(1:2)_, and **d** [Fe_3_O_4_-Fe@CNFs/Al-Fe_3_O_4_-Fe_(1:2.5)_](mailto:Fe3O4-Fe@CNFs/Al-Fe3O4-Fe(1:2.5))


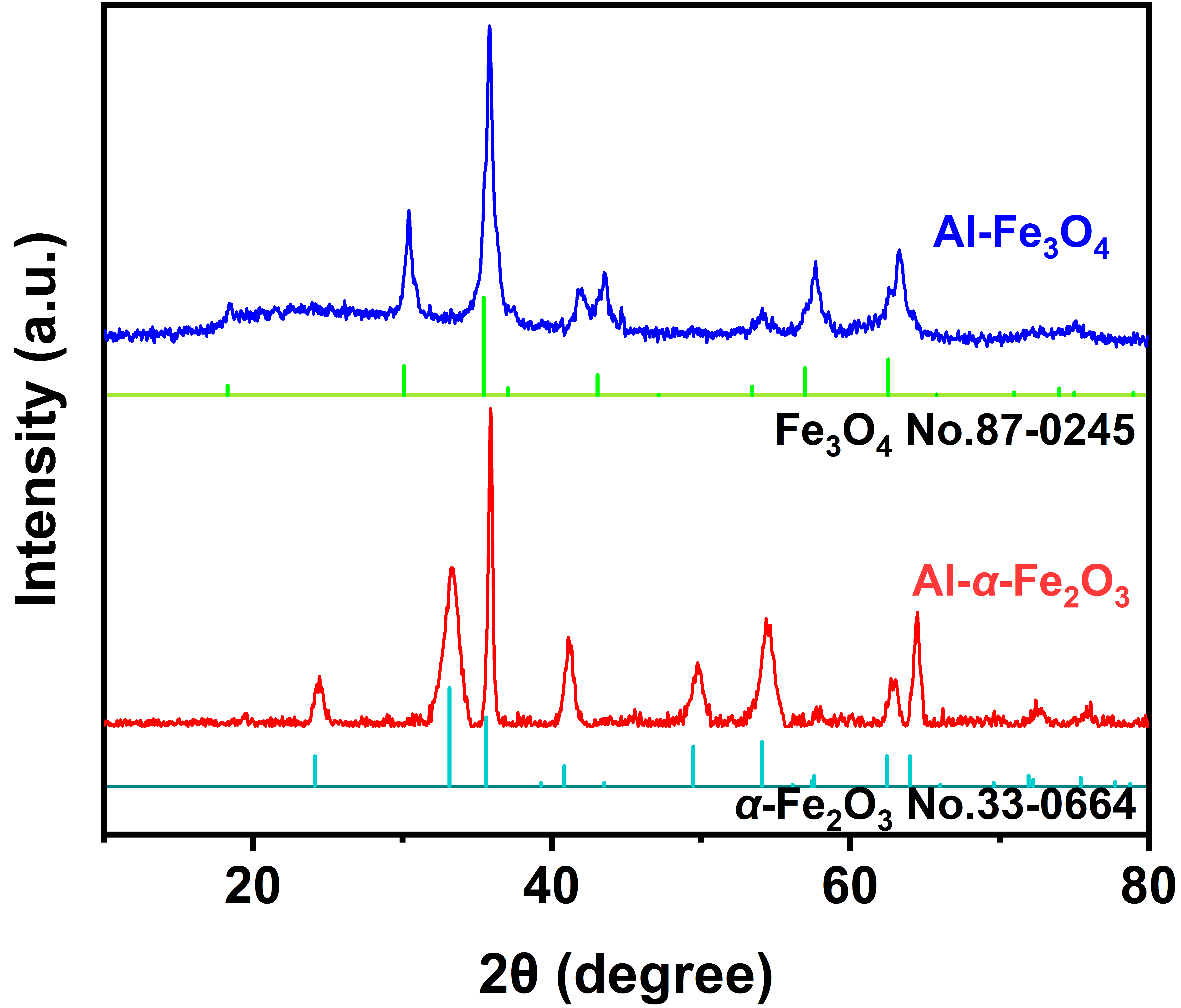


**Fig. S3** XRD patterns of Al-*α*-Fe_2_O_3_ nanosheets, and Al-Fe_3_O_4_ nanosheets


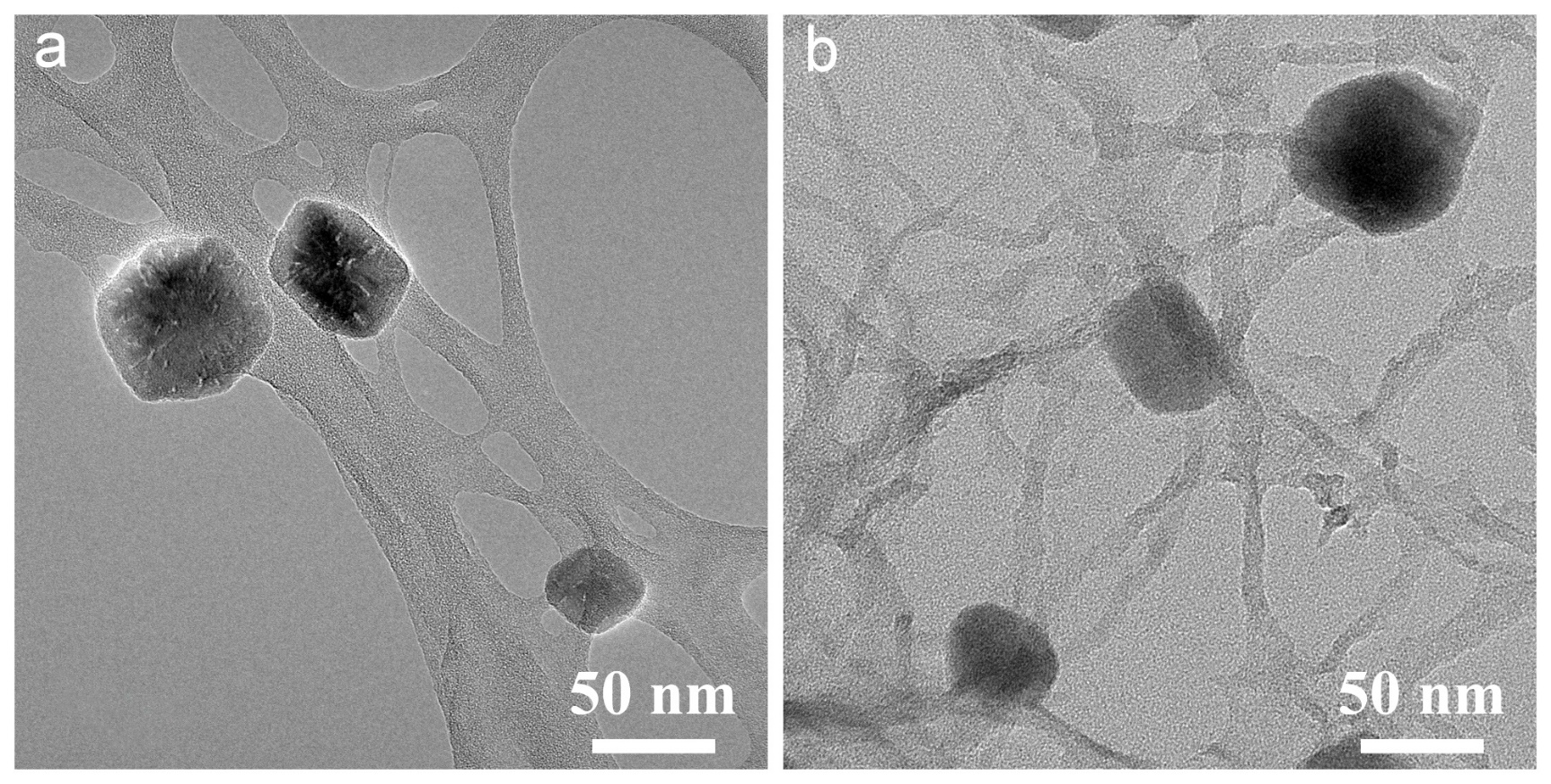


**Fig. S4** TEM images of **a** *α*-Fe_2_O_3_@ANFs and **b** Fe_3_O_4_-Fe@CNFs


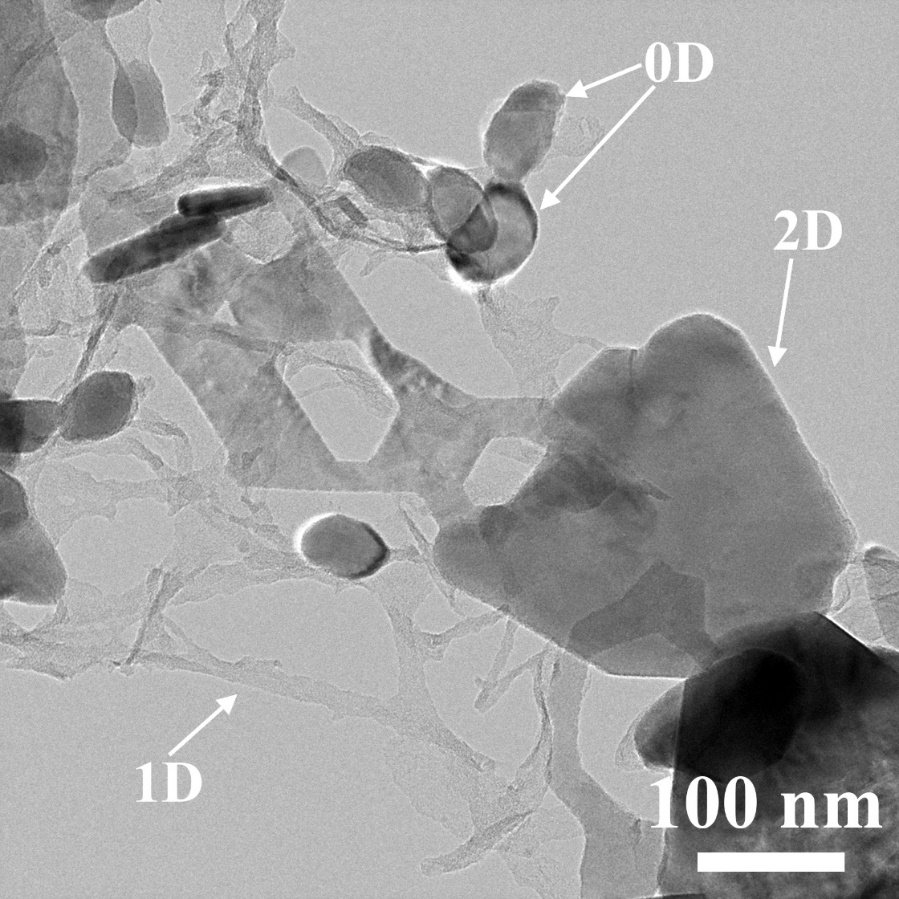


**Fig. S5** TEM image of 0D@1D/2D Fe_3_O_4_-Fe@CNFs/Al-Fe_3_O_4_-Fe


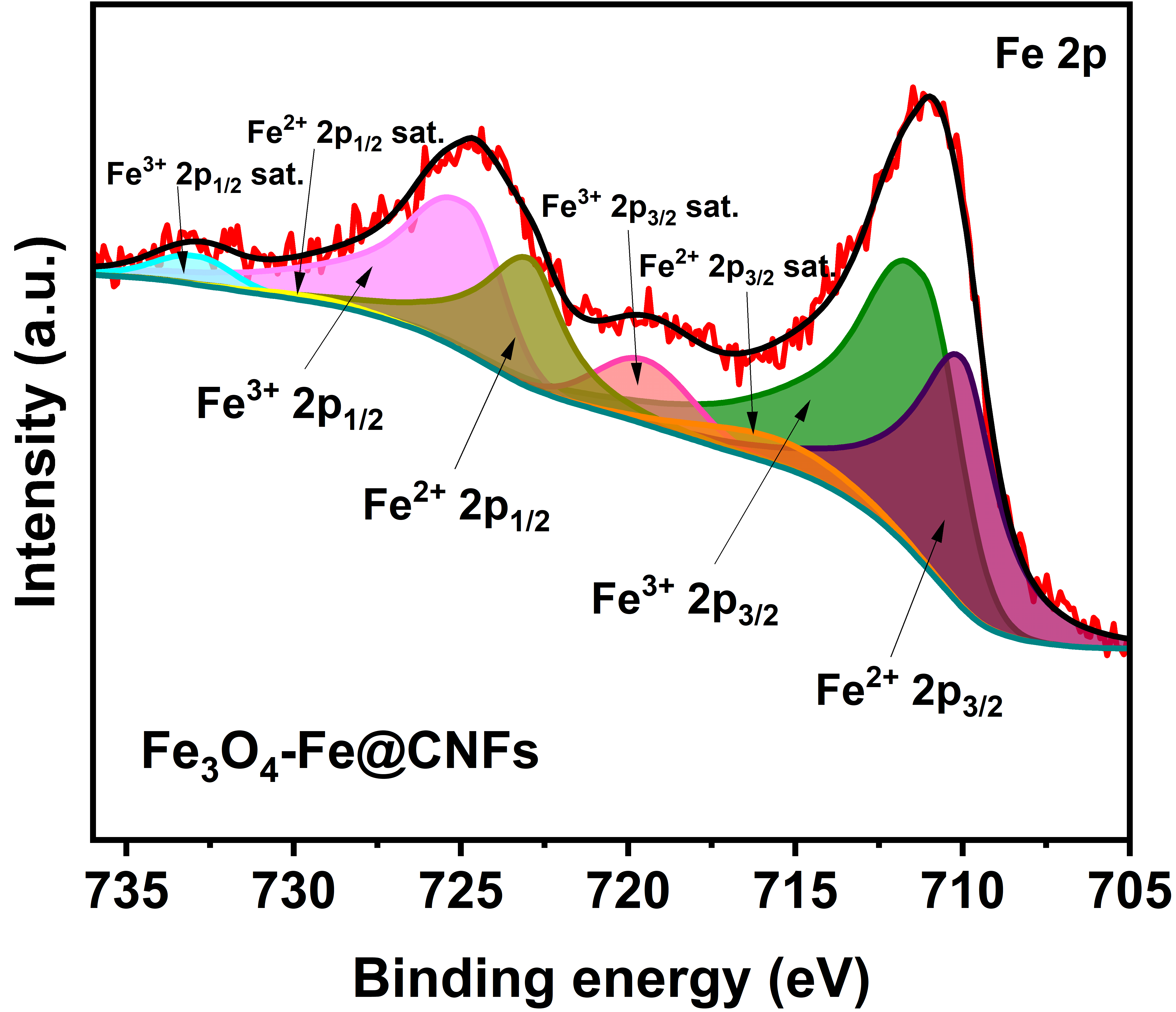


**Fig. S6** XPS spectra of Fe 2p of Fe_3_O_4_-Fe@CNFs


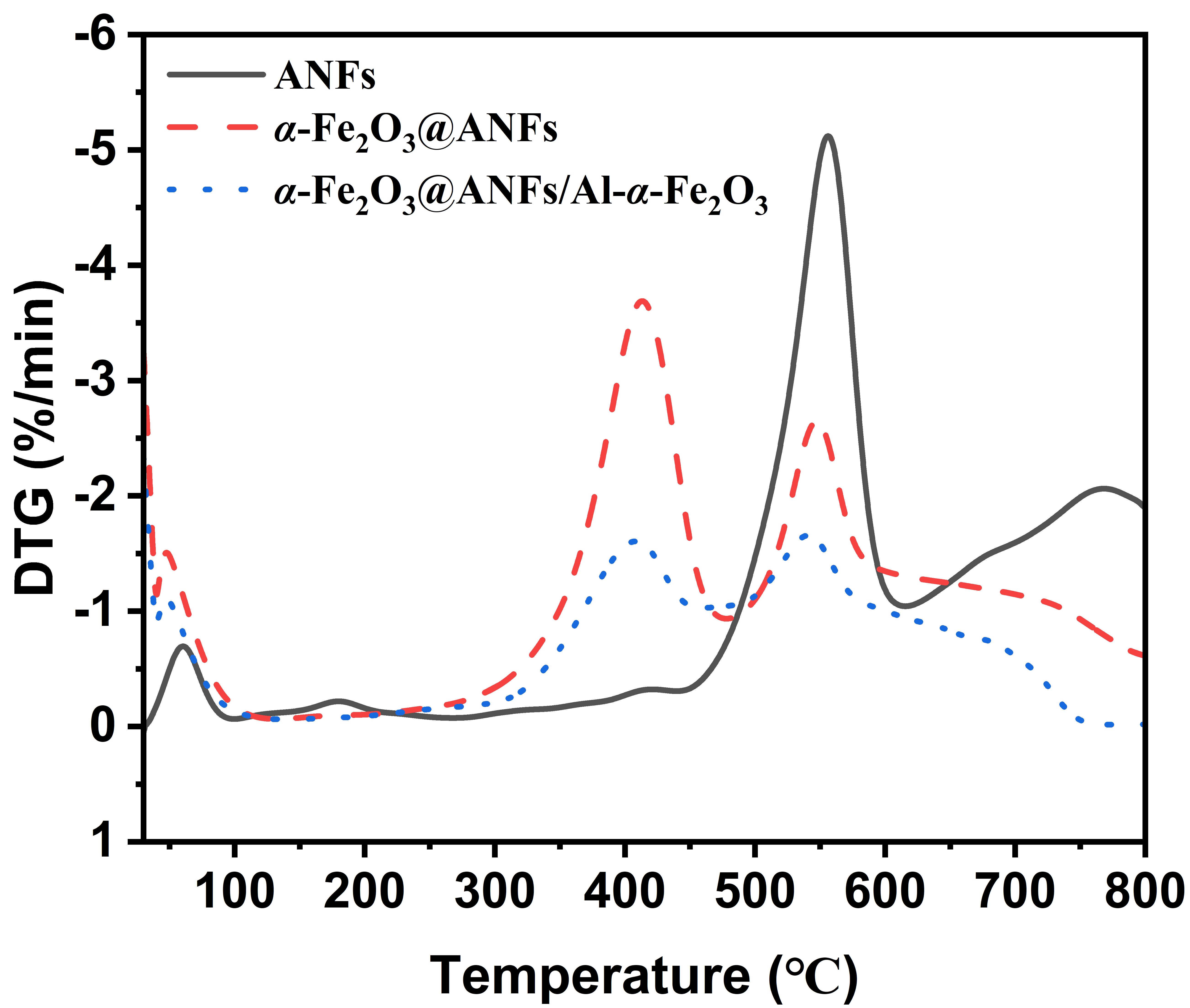


**Fig. S7** DTG curves of ANFs, *α*-Fe_2_O_3_@ANFs, and *α*-Fe_2_O_3_@ANFs/Al-*α*-Fe_2_O_3_


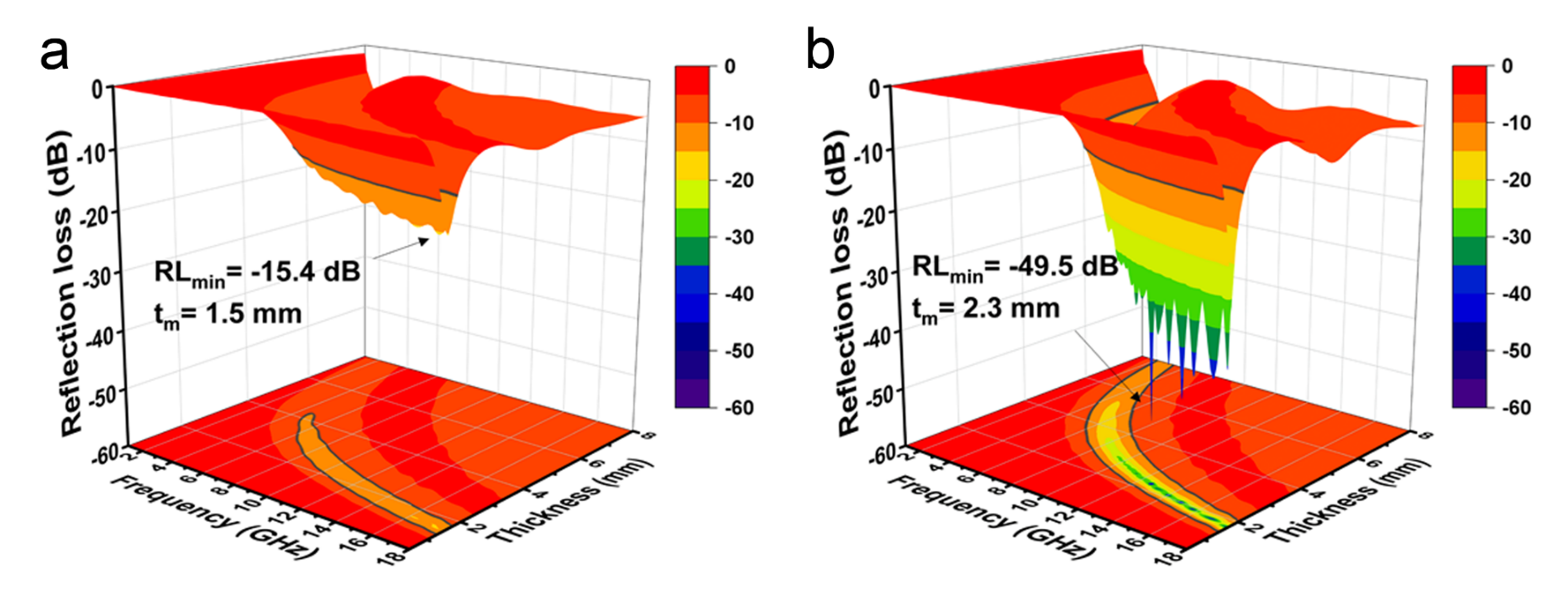


**Fig. S8** 3D reflection loss curves maps of **a** Fe_3_O_4_-Fe@CNFs/Al-Fe_3_O_4_-Fe_(1:1)_ and **b** Fe_3_O_4_-Fe@CNFs/Al-Fe_3_O_4_-Fe_(1:2.5)_

**Table S1** Comparison of electromagnetic wave absorption of the samples

| Absorbers | Component, wt | *RL*, min | | *RL*≤-10 dB | |
| --- | --- | --- | --- | --- | --- |
|  | Fe_3_O_4_-Fe : Al-Fe_3_O_4_-Fe | RL_min_  (dB) | Thickness  (mm) | EAB  (GHz) | Thickness  (mm) |
| CNFs | - | -3.8 | 0.9 | 0 | - |
| Fe_3_O_4_-Fe@CNFs | - | -24.2 | 1.9 | 5.9 | 1.9 |
| Al-Fe_3_O_4_ | - | -39.6 | 8.0 | 2.4 | 7.4 |
| Fe_3_O_4_-Fe@CNFs/Al-Fe_3_O_4_-Fe | 1: 3 | -59.3 | 4.3 | 5.6 | 2.2 |
| Fe_3_O_4_-Fe@CNFs/Al-Fe_3_O_4_-Fe_(1:2.5)_ | 1 : 2.5 | -49.5 | 2.3 | 6.1 | 2.1 |
| Fe_3_O_4_-Fe@CNFs/Al-Fe_3_O_4_-Fe_(1:2)_ | 1 : 2 | -49.5 | 2.1 | 6.4 | 2.2 |
| Fe_3_O_4_-Fe@CNFs/Al-Fe_3_O_4_-Fe_(1:1)_ | 1 : 1 | -15.4 | 1.5 | 5.7 | 1.8 |
